# How to Run Linear Mixed Effects Analysis for Pairwise Comparisons? A Tutorial and a Proposal for the Calculation of Standardized Effect Sizes

**DOI:** 10.5334/joc.409

**Published:** 2025-01-06

**Authors:** Marc Brysbaert, Dries Debeer

**Affiliations:** 1 Faculty of Psychology and Educational Sciences, Ghent University, Belgium

**Keywords:** Statistical analysis, Mathematical modelling, Face perception

## Abstract

This tutorial provides guidelines for conducting linear mixed effects (LME) analyses for simple designs, aimed at researchers familiar with t-tests, analysis of variance (ANOVA) and linear regression. First, we compare LME analyses with traditional methods when participants are the only source of random variation. We show that LME analysis is more interesting as soon as you have more than one observation per participant per condition. The second section discusses studies where both participants and stimuli are used as sources of random variation, ensuring robust generalization beyond the specific stimuli tested. In our search for standardized effect sizes, we saw that partial eta squared is even less informative for LME than for ANOVA. We present *eta squared within* as an alternative, to be used in combination with the traditional measure eta squared (also in ANOVA). To facilitate implementation, we analyze toy datasets with R and jamovi. This tutorial gives researchers a good foundation for LME analyses of simple 2 × 2 designs and paves the way for tackling more complicated designs.

Linear mixed effects analysis is increasingly used to analyze data in behavioral research (for good reasons, as we will see below). Because its introduction in psychological research is relatively recent (Baayen et al., 2008), many users are unsure how to conduct such analyses and what factors to consider. This text is intended as a practical introduction for readers familiar with basic statistical analyses, such as t-tests, analysis of variance (ANOVA) and linear regression.

Most researchers in experimental psychology associate linear mixed effects (LME) analysis with designs in which generalization across both participants and stimuli is important. As a result, they see LME analysis as a separate type of analysis, different from the basic analyses they learned in introductory statistics classes. However, LMEs are a logical extension of basic analyses and are easier to understand if we first show how an LME analysis can replace (and augment) a traditional t-test or ANOVA. Therefore, we begin by showing how LME analysis relates to traditional analyses and is a better alternative if you have more than one observation per participant per condition. This will allow us to explain advanced ideas, such as random slopes, participant centered scores, and sum contrast coding. More complex designs with both stimuli and participants as random variables are covered in Part 2.

Since statistical analyses are usually performed with software packages, we will focus on two useful tools: R and jamovi. The limitation of such a recipe approach (Schütze, 1964) is that software tools can evolve over time, making the information in this article obsolete. To address that problem, we always work with small data sets to demonstrate the output. This way, you can use the datasets to learn to work with other statistical packages if the original software is no longer available.

All the data and code for the analyses are available at https://osf.io/s3efz/. The best way to read this article is to hold the code next to you and run each analysis as it is discussed. This will give you the best understanding of how to run the analysis and how to adapt it for a new data set you want to analyze.

## 1. LME analysis as an alternative of t-tests and ANOVAs

A t-test is used to examine whether there is a statistically significant difference in the performance of two groups, or whether there is a statistically significant difference in the performance of a group of participants between two conditions. The former, which we will discuss first, is called a between-groups design, the latter a within-group design or repeated measure design.

### 1.1. A difference between two groups of participants

We start with the simplest design in behavioral research: one that compares the performance of two groups of participants. For example, we want to know whether working memory capacity is greater for 65-year-olds than for 75-year-olds.

Suppose we use an operation span task (Unsworth et al., 2005) with 20 trials. In each trial, participants must first decide whether a simple arithmetic equation is correct (e.g., is (8/2) – 1 = 1?) and then remember a word that is presented (e.g., BEAR). Trials differ in the number of equations/word sequences they contain: from 2 to 5. Each test requires the participant to memorize the words in the order presented. Participant’s score is the total number of correct words in the correct place.

To keep calculations simple, we work with a toy sample of ten 65-year-olds and ten 75-year-olds. To improve measurement accuracy, we test participants twice with one day in between, using different items. Table 1 shows the data.

**Table 1:** Toy dataset for comparison of two groups. Dependent variable is working memory capacity.


65-YEAR OLDS	day 1	day 2	75-year olds	day 1	day 2

Participant 1	42	40	Participant 11	27	27

Participant 2	23	23	Participant 12	35	37

Participant 3	44	46	Participant 13	43	43

Participant 4	20	20	Participant 14	51	49

Participant 5	43	47	Participant 15	19	25

Participant 6	37	37	Participant 16	52	50

Participant 7	48	46	Participant 17	34	34

Participant 8	53	53	Participant 18	24	26

Participant 9	50	52	Participant 19	35	35

Participant 10	33	33	Participant 20	23	21


#### 1.1.1. Analysis with t-test, ANOVA, and regression

The easiest-to-understand test for comparing two groups is the t-test. It basically compares the difference in mean score between the two groups and looks at how big this difference is relative to the variability in each group.

**Figure 1:**
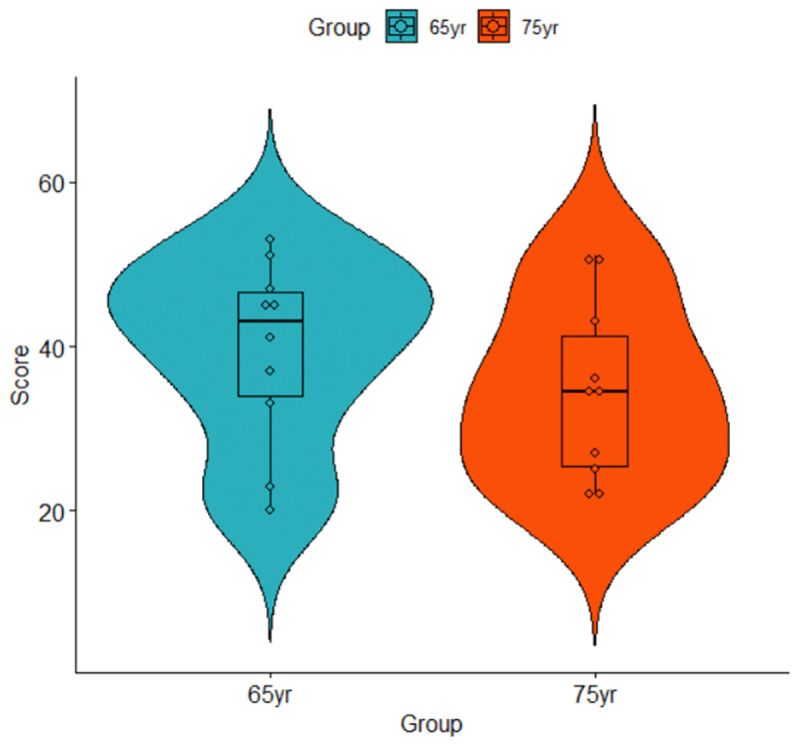
Violin plot of the data listed in Table 2.

The t-test requires one observation per participant. This can pose a challenge for beginners as experimental data sets often contain several observations per participant. This is also true for the data in Table 1, where there are two observations per participant. A simple solution is to take the mean value per participant, as shown in Table 2.^1^ Table 2 also includes the mean (M) and standard deviation (SD) of each group. This shows that the 65-year-olds generally had higher memory scores (M = 39.5) than the 75-year-olds (M = 34.5), a difference that can also be seen in Figure 1. We want to know whether the difference is statistically significant.

**Table 2:** Data of the toy dataset after averaging the scores of day 1 and day 2.


65-YEAR OLDS	SCORE	75-year olds	SCORE

Participant 1	41	Participant 11	27

Participant 2	23	Participant 12	36

Participant 3	45	Participant 13	43

Participant 4	20	Participant 14	50

Participant 5	45	Participant 15	22

Participant 6	37	Participant 16	51

Participant 7	47	Participant 17	34

Participant 8	53	Participant 18	25

Participant 9	51	Participant 19	35

Participant 10	33	Participant 20	22

	M = 39.5		M = 34.5

	SD = 11.23		SD = 10.78


Because the data set is small, we can calculate the t-test by hand (Brysbaert, 2020), using the following equation (N = number of participants in each group):


\[
t\,{ = }\frac{{{M_{65yr}}-{M_{75yr}}}}{{\sqrt {\frac{{SD_{65yr}^2}}{{{N_{65yr}}}}{ + }\frac{{SD_{75yr}^2}}{{{N_{75yr}}}}} }}{ = }\frac{{39.5-34.5}}{{\sqrt {\frac{{{{11.23}^2}}}{{10}}{ + }\frac{{{{10.78}^2}}}{{10}}} }}{ = }\,1.02
\]

To interpret the t-value, we need the degrees of freedom, which for a between-groups t-test is N_65yr_ – 1 + N_75yr_ – 1 = 10 – 1 + 10 – 1 = 18. This gives us a p-value of t(18) = 1.02, p = .323, two-tailed,^2^ meaning that the difference is not significant at the .05 level.

An easier way to get the result is to use existing software. There are plenty of software packages doing the calculations for us. One of the richest is R. The t-test is part of many libraries in R and it is also part of the base packages, meaning that we do not need to install or load a package.

The standard way to set up the t-test for independent samples in R is to input the data in long format, with each observation on a separate row, as shown in Table 3. You can do this in R (see code below), but also in Excel. We will need the long format for most of the article.

**Table 3:** Long format input to run the t-test in R and in jamovi. Make sure you have 20 lines (2 groups * 10 participants in each group).


PARTICIPANT	GROUP	SCORE

Participant 1	65yr	41

Participant 2	65yr	23

Participant 3	65yr	45

Participant 4	65yr	20

Participant 5	65yr	45

Participant 6	65yr	37

Participant 7	65yr	47

Participant 8	65yr	53

Participant 9	65yr	51

Participant 10	65yr	33

Participant 11	75yr	27

Participant 12	75yr	36

Participant 13	75yr	43

Participant 14	75yr	50

Participant 15	75yr	22

Participant 16	75yr	51

Participant 17	75yr	34

Participant 18	75yr	25

Participant 19	75yr	35

Participant 20	75yr	22


Once you have imported the data set in the right format in R,^3^ you can run the following command:

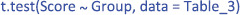

The output gives you all the information you need, except that the better^4^ Welch t-test is used by default instead of the original Student t-test. Notice that the p-values is for a two-sided test as default. This will be case for all the packages we describe.

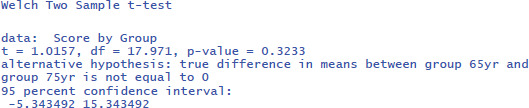

The p-value indicates the probability that we would observe this particular set of data if the null hypothesis were true. It can be seen as a quantification of the evidence against the null hypothesis: the smaller the p-value, the more evidence against the hypothesis of equal means. For the data set we analyzed, the evidence is not strong enough to assume that the null hypothesis is wrong, as the p-value is not smaller than .05. Because the p-value depends both on the difference between the groups, as well as on the sample size, it cannot be interpreted as a measure of effect size.

The most informative effect size consists of the mean and the standard deviation (SDs) in each condition (Baguley, 2009; Pek & Flora, 2018; Rights & Sterba, 2019; Vanhove, 2015). Therefore, it is crucial to always present this information in a clear and concise table within your statistical analysis section.

Additional information is provided by a *standardized* effect size. This metric expresses the size of a difference in relative terms. It allows you to measure the impact of your finding compared to findings from other studies, even if those studies worked with different materials and variables.

One of the most used standardized effect sizes in psychology is Cohen’s d. This is the difference between the means divided by the average standard deviation of the conditions.

You obtain Cohen’s d if you install and load the R package “effectsize” (Ben-Shachar et al., 2023). You do this as follows:

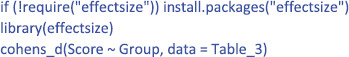

This gives the output:

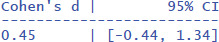

The outcome not only tells you that the standardized effect size is d = .45, but also that its 95% confidence interval ranges from d = –.44 to d = 1.34. This basically means that with a sample size of 10 participants per group, you may obtain d-values going from a medium effect in favor of 75-year-olds to a very large effect in favor of 65-year-olds. This is a clear sign that your study is not very informative about the effect size at the population level, because of the small sample sizes (Brysbaert, 2019, 2021).

**Figure 2:**
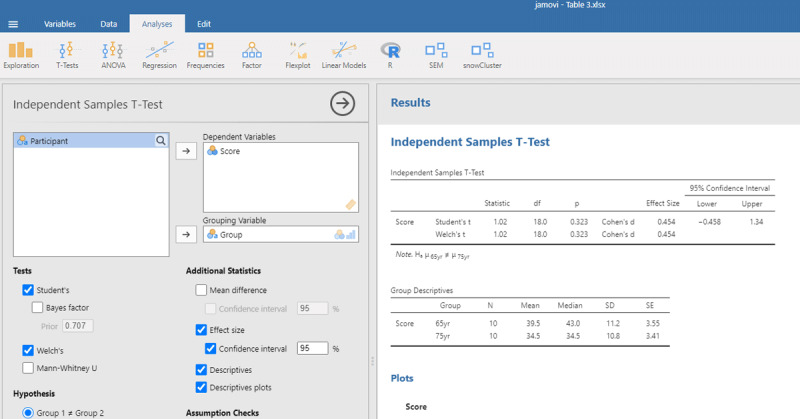
jamovi output for the t-test of working memory capacity between two age groups.

We can also calculate the t-test with a freely available, novice-friendly statistical package, such as jamovi (The jamovi project, 2023). We assume that entering data and clicking on buttons is clear for everyone with minimal computer knowledge (you also find all jamovi files with data entered and the right buttons clicked at https://osf.io/s3efz/; if you have jamovi installed on your computer, simply start the program and open the files). The output is shown in Figure 2.

The same dataset can be analyzed with an analysis of variance or ANOVA. For the example, we need an ANOVA with Age as the only between-subjects independent variable.

In R you get the outcome with the following commands:

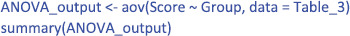

It gives the output (see Brysbaert, 2020, for a discussion of how ANOVA tables are calculated and what the various entries mean):

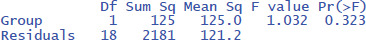

**Figure 3:**
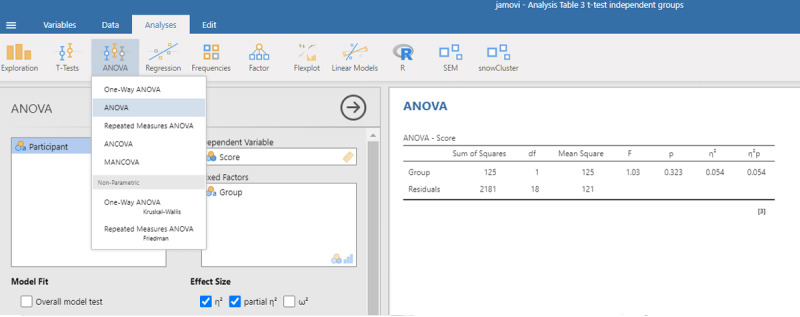
Output jamovi ANOVA between-groups analysis working memory capacity.

Figure 3 gives the output of jamovi.

The p-values of the F-test in ANOVA and the t-test are the same, because the F and t-values are mathematically related to each other. When there are only two conditions, the F-value of the ANOVA equals the square of the t-value from the t-test [i.e., F(1,18) = 1.032 = t²(18) = 1.016²].

ANOVAs give us another measure of standardized effect size. It is the percentage of variance accounted for by the independent variable, called eta-squared (η²). In the jamovi output we see that the Group difference accounted for 5.4% of the variance between participants (η² = .054). To get the η² value in R, use the following commands:^5^

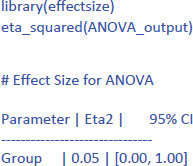

The notion “percentage of variance explained” is more familiar in linear regression analysis. There it is customary to see how much variance is explained by the predictors and call it R². As it happens, we can analyze our dataset with a linear regression analysis as well, using Group as a categorical (or nominal) variable meaning that there is no magnitude associated with it.

The R-commands are shown below. They are rather simple because on the basis of the participant names R automatically detects that the Group variable is a categorical variable.

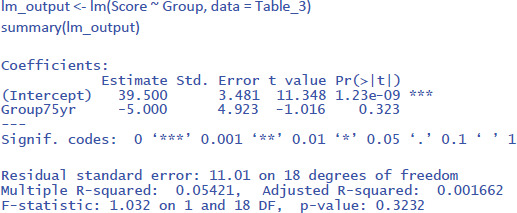

The output of the linear regression gives the same information as the t-test or the ANOVA: The effect of Group is not significant (t = –1.02, p = .323) and accounts for 5.4% of the variance in the observations (R² = .05421 = η²).

**Figure 4:**
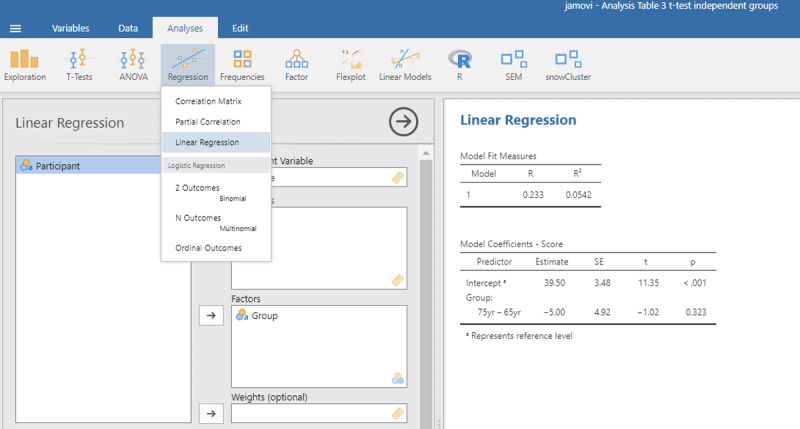
Output jamovi linear regression analysis of between-groups example working memory.

To run the analysis in jamovi, we have to indicate that Group is a categorical variable by putting it under Factors and not under Covariates (which are continuous variables, like the age of the participants in years).^6^ Figure 4 shows the output.

Now that we know what our data look like in traditional analyses, we can compare them to a linear mixed effects (LME) analysis. The outcome will not be exactly the same, because in the traditional analyses we took the average performance of the participants, whereas in LME we take both measurements of each person into account. However, the pattern of findings should be very similar, because in our example we did not introduce big differences between the two measurements.

#### 1.1.2. Analysis with LME

An advantage of LME over a t-test is that we do not have to average the data for each participant. We can just work with the raw values from Table 1. Thus, we do not have to calculate Table 2. This is not only convenient, but also better from a statistical point of view, because we are considering the full variability in the data set. When we average the scores of day 1 and day 2 for a t-test or ANOVA, we assume that there are no differences between these scores (or at least that they are not important for the question we are investigating).

A new element in LME analysis is that we must make a distinction between fixed effects and random effects. Fixed effects refer to the variables we manipulate and for which we are interested in the levels tested. In our example, that is the age of the participants (two groups). Random effects refer to variables that also influence the data, but for which we have no specific predictions or expectations. In our example, these are the participants. We accept that participants differ from each other in level of performance: Some participants have a higher working memory capacity than others. We have no further explanation for these differences. We simply assume that the participant scores are a random sample from a normally distributed population.

The difference in performance level between participants is called the ***participant intercept***. To estimate the participant intercept, we need at least two observations per participant (if you have only one, the analysis reduces to a t-test, ANOVA, or regression). In an LME analysis of two independent groups we assume that the data are a combination of the fixed effect we manipulated (age group), a random intercept (performance level) for each participant, and residual measurement error.

The R package mostly used for LME is lme4 (Bates et al., 2022), often accessed through the lmerTest package (Kuznetsova et al., 2022), which provides p-values. For this analysis, we need a new input file in long format, which contains all the information from Table 1. It looks like Table 4.

**Table 4:** Outline of the input file for an LME analysis in R and jamovi (total number of data lines is 40: 20 participants * 2 days).


PARTICIPANT	GROUP	TIME	SCORE

Participant 1	65yr	day1	42

Participant 1	65yr	day2	40

Participant 2	65yr	day1	23

Participant 2	65yr	day2	23

…			


Then you can run the following R code

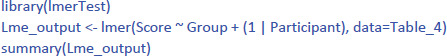

The code *(1 | Participant)* indicates that we add random intercepts for the participants.

The R commands will provide you with the following output:^7^

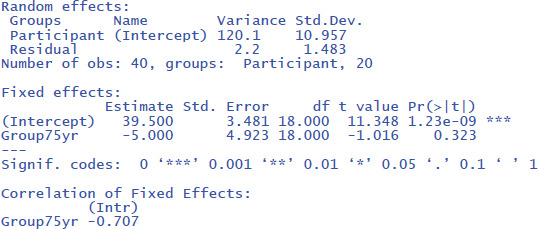

From the output you can see that the critical test of the fixed effect Group is very similar to the one we found in the t-test analysis (t(18) = –1.02, p = .323). Under random effects, you also see the large variability due to differences in intercepts among participants (StdDev = 10.957). In fact, this estimate is close to the standard deviation observed in the raw scores (Table 2). This is because there are no other big sources of variance (differences between groups, days of testing).

The following command from the package effectsize can be used to obtain the standardized effect size η²:^8^

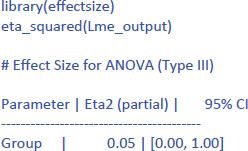

**Figure 5:**
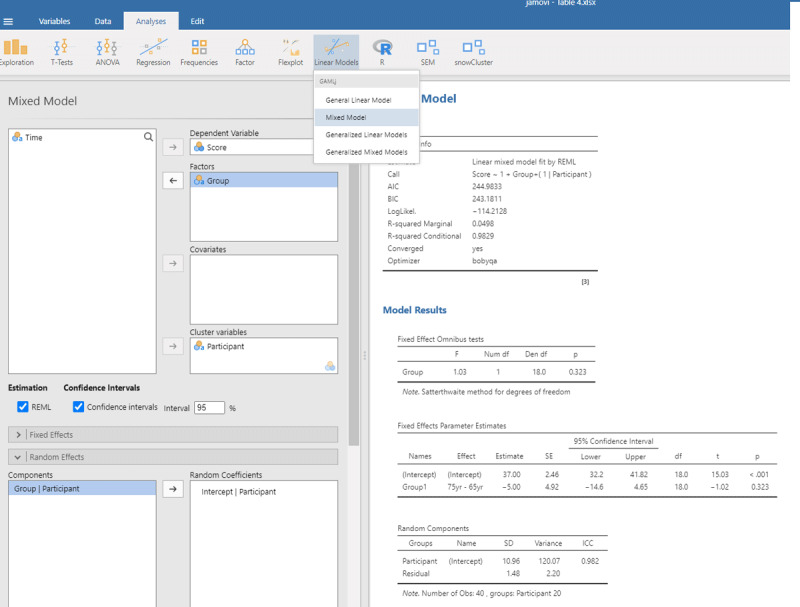
Output jamovi LME analysis of between-groups example working memory.

Jamovi gives the same output in an intuitive way (Figure 5). To obtain it, upload the package gamlj (Gallucci, 2022) from the jamovi library and run the mixed model, as shown below. You must enter Score as the dependent variable, Group as a categorical factor, and Participant as a cluster variable. In addition, under Random Effects, you must select the intercept of the participants.

If we look at the output, we see that the t- and p-values are reassuringly the same as before (t = 1.02, p = .323). We also see an entry called R-squared Marginal (=.0498). This values corresponds to η² and R² in the analyses we did before. So, all necessary information is present in jamovi.

However, there is an important difference between the default choices made in gamlj and in lme4. This can be seen in the value of the intercept in the fixed effects table. In gamlj, the intercept is 37, whereas in lme4 and lmerTest it is 39.5. In both analyses, the difference between the groups is –5. What is going on here? And why is it important?

The R packages lme4 and lmerTest use **dummy coding** as the default (like R in general). They assign 0 to the first condition (here 65yr) and 1 to the second condition (here 75yr). As a result, the mean score of the first condition corresponds to the intercept of the model (M_65yr_ = 39.5 + 0 * difference between conditions = 39.5) and the mean score of the second condition corresponds to the intercept plus the difference between the conditions (M_75yr_ = 39.5 + 1 * difference between conditions = 39.5 – 5 = 34.5).

In contrast, the jamovi package gamlj uses **sum coding** as default with contrast weights of –.5 and +.5. As a result, the intercept corresponds to the overall mean of the data set (i.e., (39.5 + 34.5)/2 = 37). The mean of condition 65yr corresponds to the intercept – 0.5 times the difference between conditions (= 37 + (–.5) * (–5) = 39.5) and the mean of condition 75yr corresponds to the intercept + 0.5 difference between conditions (= 37 + (+.5) * (–5) = 34.5).

You can get the same R output in gamlj by changing the default value of “Factors coding” from “simple” to “dummy”. You can also change the R code to use sum coding instead of dummy coding, as we will see below.

The distinction between dummy coding and sum coding may seem trivial now, but later in this article we will see that sum coding is needed to get useful interpretation for the main effects when your model includes an interaction between the independent variables!

LME offers a further advantage over traditional methods in dealing with missing data. LME can efficiently deal with missing values, provided they are not excessive and randomly distributed across conditions, so LME results obtained with missing data are very similar to those from full data sets. In contrast, t-tests and ANOVAs have more stringent requirements and require observations in all cells. This often leads to the exclusion of participants with missing data or the need for imputation techniques, as described by Austin et al. (2021).

Hopefully our example has shown you how LME relates to simpler tests. Three advantages of LME are: (1) you do not need to calculate means (you can simply use all values in long format like in Table 4), (2) the model deals efficiently with missing observations, and (3) the model can be extended to more than one random variable, as we will see in Part 2.

First, we will discuss two more designs to set the stage.

### 1.2. A difference between two related or paired conditions

A between-groups design is nice, because the observations are independent, which simplifies the calculations. However, such a design generally requires many participants to have decent power (Brysbaert, 2019). Power is higher if participants participate in both conditions, as this eliminates performance differences between participants. Observations coming from the same participants are called related, paired, or repeated measures (feel free to make your choice).

To illustrate the various analyses, we again make use of a toy data set. Suppose the 65-year-olds from Table 1 were part of a longitudinal study and were retested 10 years later, when they were 75 years old. Again, two parallel working memory capacity tests were administered on two separate days. Table 5 shows the data obtained.

**Table 5:** Longitudinal data of a group of participants tested twice (day 1 & 2) at different ages (65 & 75 years). Dependent variable is working memory capacity.


PARTICIPANT	65yr day 1	65yr day 2	75yr day 1	75yr day 2

Participant 1	32	30	27	27

Participant 2	38	42	35	37

Participant 3	42	38	43	43

Participant 4	57	55	51	49

Participant 5	24	30	19	25

Participant 6	57	49	52	50

Participant 7	37	39	34	34

Participant 8	33	33	24	26

Participant 9	40	38	35	35

Participant 10	22	20	23	21


#### 1.2.1. Analysis with t-test and ANOVA

**Figure 6:**
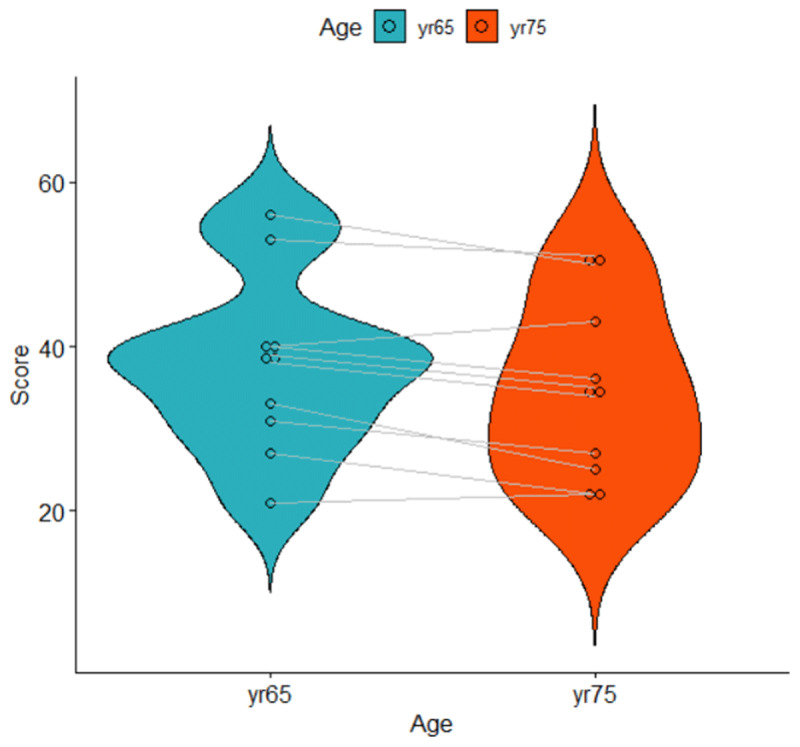
Violin plot of the data shown in Table 6. Lines represent related observations.

For a t-test of paired observations, we need (1) a single score per participant per condition, and (2) the difference between the conditions for each participant. First, we take the mean of each participant at each age. Second, for each participant, we compute the difference between the two ages. Note that by computing the differences, performance difference between participants are removed. Table 6 and Figure 6 show what the data look like. They show that the average memory score of the participants was higher when they were 65 years old (M = 37.8) than when they were 75 years old (M = 34.5). Is the difference statistically significant?

**Table 6:** Table for a t-test repeated measure example working memory.


PARTICIPANT	65yr	75yr	diff

Participant 1	31	27	4

Participant 2	40	36	4

Participant 3	40	43	–3

Participant 4	56	50	6

Participant 5	27	22	5

Participant 6	53	51	2

Participant 7	38	34	4

Participant 8	33	25	8

Participant 9	39	35	4

Participant 10	21	22	–1

	M_65_ = 37.8	M_75_ = 34.5	M_diff_ = 3.3

	SD_65_ = 10.76	SD_75_ = 10.78	SD_diff_ = 3.23


The t-test for related or paired observations is calculated as follows (N = number of participants):


\[
t\,{\mathrm{ = }}\frac{{{M_{diff}}}}{{S{D_{diff}}/\sqrt N }}{\mathrm{ = }}\frac{{3.3}}{{3.23/\sqrt {10} }}{\mathrm{ = }}\,3.23
\]

The test has N – 1 = 9 degrees of freedom and is significant: t(9) = 3.23, p = .010, two-tailed. The test is significant despite the small number of participants, because the effect size is large. We can calculate the effect size as follows:


\[
{d_z}\,{ = }\,\frac{{{M_{diff}}}}{{S{D_{diff}}}}\,{ = }\,\frac{{3.3}}{{3.23}}\,{ = }\,1.02
\]

The effect size expresses the expected average difference between the two conditions for a participant, relative to the variability in difference scores. It is traditionally called d_z_.

The reason why the effect size and t-value are large is that most participants have greater working memory capacity at age 65 than at age 75. Therefore, the standard deviation of the difference scores is small (Table 6).

The effect size d_z_ builds on the assumption that differences in overall performance between participants are not important. Only the differences between conditions matter. Note that this assumption was the reason why a repeated-measures design was used in the first place. In some situations, however, it is interesting to also compare the condition effect to the overall variability in the sample. This is the case, for example, when we conduct a meta-analysis over studies that sometimes manipulated the variable within participants and sometimes between participants. So, we can define a second effect size, d_av_, which takes all variability into account. Because this effect size includes all variability, the value of d_av_ is very similar to the d-value we obtained in our previous analysis of independent samples:


\[
{d_{av}}\,{ = }\,\frac{{{M_{65}}-{M_{75}}}}{{\sqrt {\frac{{SD_{65}^2\,{ + }\,SD_{75}^2}}{2}} }}\,{ = }\,\frac{{37.8\,-\,34.5}}{{\sqrt {\frac{{{{10.76}^2}\,{ + }\,{{10.78}^2}}}{2}} }}\,{ = }\,0.31
\]

The difference between d_z_ and d_av_ is a headache for researchers running meta-analyses on topics in which both between-groups and repeated measures designs are used, because the statistics provided in the manuscripts often do not suffice to calculate both. You can help them by including this information in your text (see Fernández-Castilla et al., 2024, for guidelines about how to report data so that they can easily be included in meta-analysis).^9^

To run the t-test for related samples in R or jamovi, we use the format of Table 7.

**Table 7:** Input for R and jamovi analysis of t-test related samples.


PARTICIPANT	yr65	yr75

Participant 1	31	27

Participant 2	40	36

Participant 3	40	43

Participant 4	56	50

Participant 5	27	22

Participant 6	53	51

Participant 7	38	34

Participant 8	33	25

Participant 9	39	35

Participant 10	21	22


The R code then is the following (if you have named your dataset Table_7).

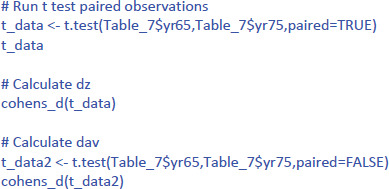

This gives the following output:

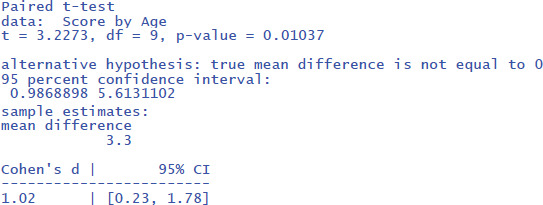

The output tells us that the confidence interval of the effect size goes from d = .23 to d = 1.78; that is from a small effect size to a very large effect size. This informs us that the uncertainty about the effect size is high because of the small sample size used.

**Figure 7:**
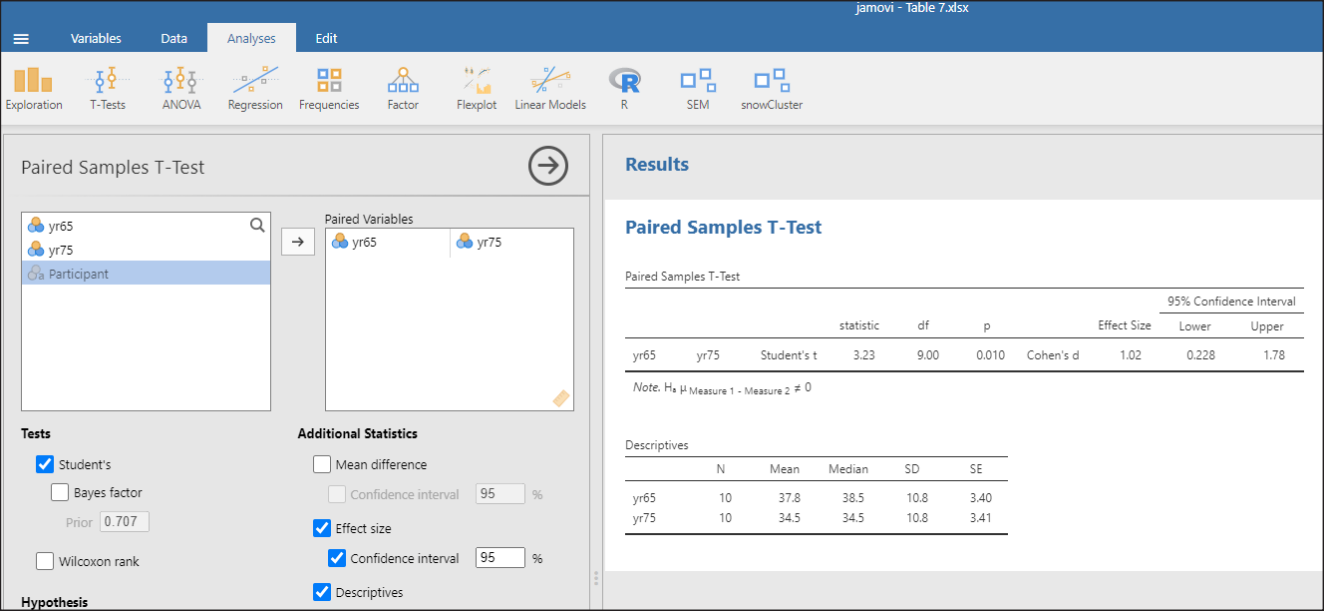
Output jamovi t-test longitudinal study working memory.

The jamovi output for the t-test is shown in Figure 7.

For the present discussion, it is important to understand what is done in a t-test for related conditions. First, the overall differences in performance between participants are left out of consideration by using the difference scores. Second, the t-test looks at whether the average difference between conditions differs from zero given the *variability in difference scores*. The latter is an important insight, because it means we not only consider the overall performance of the participants (their intercepts) but we also consider how participants vary in their differences or change between conditions. The change or difference between conditions is also called the **slope**. So, in LME we will have to enter both random intercepts and random slopes by participants.

Before discussing the LME analysis, we first analyze the data of Table 7 with an ANOVA for repeated measures to get a better feel for the data and the various statistics that can be calculated. In R we can do this with the code below (which incidentally shows that the basic R code is not always easy to remember; there are R packages that are more user-friendly):

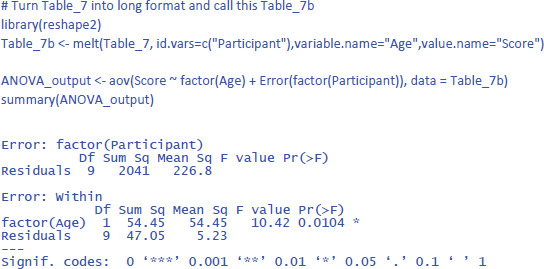

**Figure 8:**
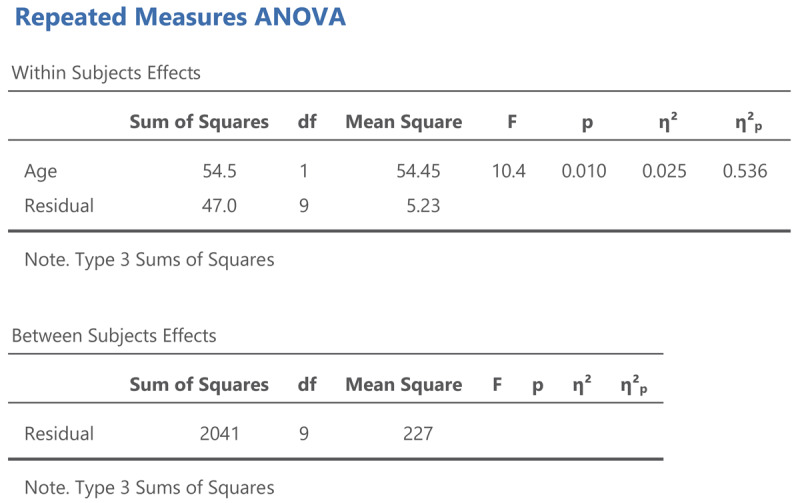
Output jamovi ANOVA longitudinal study working memory.

The output of the same analysis in jamovi is shown in Figure 8.

The output of an ANOVA for repeated measures shows that a difference is made between within-subjects effects and between-subjects effects. The variance between subjects refers to the differences in overall performance between the participants (i.e., the participants’ intercepts). The variance within subjects refers to the age effect and the variability in the age effect (the participants’ slopes).

The jamovi output also gives two standardized effect sizes: η² (eta squared) and η²_p_ (eta squared partial).^10^ η² tells us how much of the total variance is explained by Age. This is only 2.5%. You obtain this value when you divide the Age sum of squares by the total of all three sums of squares in the ANOVA table, as can be seen in the equation below:


\[
{\eta ^2}\,{ = }\,\frac{{54.5}}{{54.5\,{ + }\,47.0\,{ + }\,2041}}\,{ = }\,.025
\]

The value of η² is low because participants differ a lot in working memory capacity, as can be seen in Figure 6. This variability is captured by the between subjects sum of squares, which amounts to 2041, much more than the variability associated with the Age effect (54.5) and the variability in Age effect between participants (47.0).

Here, to some extent, η² is not very interesting, because we used a repeated measures design, exactly to deal with the problem that people differ in working memory capacity. We retested a group of participants 10 years later to see whether most of them would show a decline, *independent of the capacity they had at the start of the study*. For this, η²_p_ is more informative. You obtain it by dividing the Age sum of squares by all variance within-subjects:


\[
\eta _p^2\,{ = }\,\frac{{54.5}}{{54.5\,{ + }\,47.0}}\,{ = }\,.536
\]

The value η²_p_ gives the percentage of variance in the difference scores (or slopes) explained by Age. It tells us that more than half of the variability is accounted for by Age. This is a very big percentage, as psychological variables rarely explain more than 10% of variance (Bosco et al., 2015; Lovakov & Agadullina, 2021; Schäfer & Schwarz, 2019; Sladekova et al., 2023).

Further interesting is that η² gives the same information as d_av_, whereas η²_p_ gives the same information as d_z_. Indeed, you can translate one effect size into the other:^11^


\[
\begin{array}{l}
{d_{av}}\, \approx \,2\,*\,\sqrt {\frac{{{\eta ^2}}}{{1-{\eta ^2}}}} \, = \,2\,*\,\sqrt {\frac{{.025}}{{1-.025}}} \, = \,.32\\
{d_z}\, \approx \,\sqrt {\frac{{\eta _p^2}}{{1\,-\,\eta _p^2}}} \, = \,\sqrt {\frac{{.536}}{{1\,-\,.536}}} \, = \,1.07
\end{array}
\]

You may wonder why the equation of d_av_ includes a multiplication by 2, whereas that of d_z_ does not. This is because two independent distributions are compared in a between-groups comparison (the distribution of the 65yr and that of the 75yr), whereas only one distribution is considered in repeated measures (that of the difference scores between 65yr and 75yr). Still, the different relationship between d_av_ and η² and between d_z_ and η²_p_ is the origin of many misunderstandings in the literature. It is also the reason why a d effect size does not work in LME, because an effect can be between-conditions for one random variable but within-conditions for another random variable, as we will see shortly. Therefore, we argue it is better to stick to eta squared in LME rather than relative differences in means, just as we work with R² in linear regression.

You can get the values of η² and η²_p_ in R via the package effectsize:

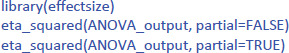

#### 1.2.2. Analysis with LME

Now that we have covered the basics of an analysis for repeated measures, let’s see what the LME analysis looks like. Remember that for this analysis we look at the entire dataset, not at the averages of two measurements (days tested). So, there will be some more variance in our dataset. Also remember that we must include both random intercepts and random slopes for the participants. We will start with the gamlj analysis in jamovi, as here we can use simple clicks to see what comes out. The input for LME must be in long format, as shown in Table 8.

**Table 8:** Long format version of Table 5, needed as input for LME analysis related samples (must contain a total of 40 data rows: 10 participants * 2 ages * 2 days tested).


PARTICIPANT	AGE	DAY	SCORE

Participant 1	yr65	day1	32

Participant 2	yr65	day1	38

Participant 3	yr65	day1	42

Participant 4	yr65	day1	57

Participant 5	yr65	day1	24

Participant 6	yr65	day1	57

Participant 7	yr65	day1	37

Participant 8	yr65	day1	33

Participant 9	yr65	day1	40

Participant 10	yr65	day1	22

Participant 1	yr65	day2	30

…			


**Figure 9:**
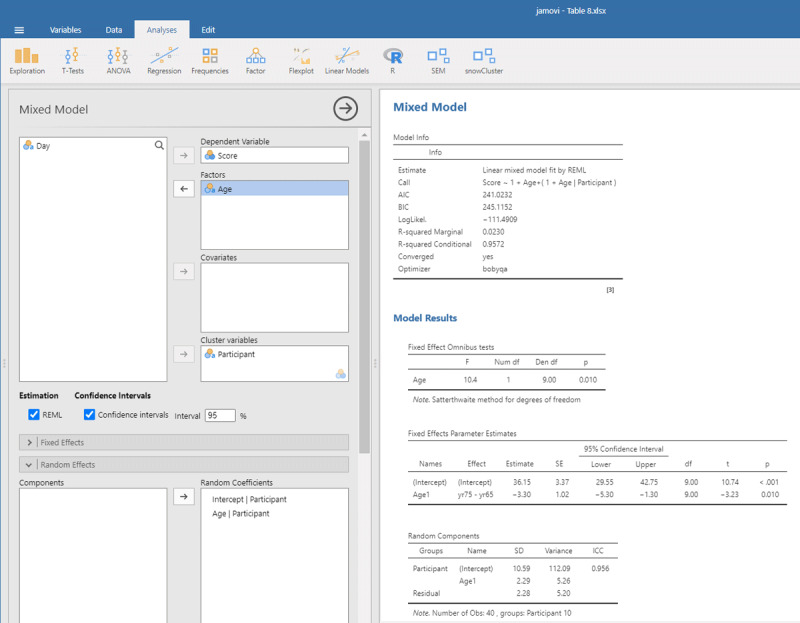
Output jamovi LME analysis longitudinal study working memory.

We open mixed model in gamlj (under the heading Linear Models in jamovi). We enter Score as the dependent variable and Age as the independent variable. Because age is a categorical variable (consisting of two separate age groups), we put it under factor (and not under covariates). In addition, we must enter that the intercept and the Age slope are random variables by participants (each participant has their own performance level and shows variability in the size of the Age effect). If we do everything correctly, we get the output shown in Figure 9.

Reassuringly, the t-value for Age is similar to the t-test we ran: t(9) = –3.23, in line with the fact there were few differences between the two days of testing. Notice that the sign of the t-test is negative here whereas it was positive before. This is because the sign depends on how the conditions are compared to each other (65yr–75yr or 75yr–65yr). Therefore, it is necessary to always provide information about the means and SDs of the conditions when you report the outcome of a statistical analysis so that readers can check that you are drawing the right conclusions!

Further notice that the jamovi output gives information about the random effects. These tell you that the variability in intercepts is much larger than the variability in slopes and the residual noise of measurements.^12^ Because jamovi coded Age as –.5 and .5, SD of the random slope can be interpreted as the variability in the score difference between the two age groups.

The jamovi output further tells us that R²_marginal_ = .0230, which is lower than the η² = .025 we obtained in the ANOVA. This is because the differences between the measurements are added to the total variability in the dataset. As a matter of fact, this is better because if we want to know the percentage of *total* variance explained, it is not a good idea to average values. Averaging reduces the variance observed in the original scores, so that the value of η² only applies to studies in which the same number of observations have been averaged (Brysbaert & Stevens, 2018).

To conduct the LME analysis in R, we use the code lines below. The instruction (Age | Participant) indicates that both random intercepts and random slopes must be included for participants.

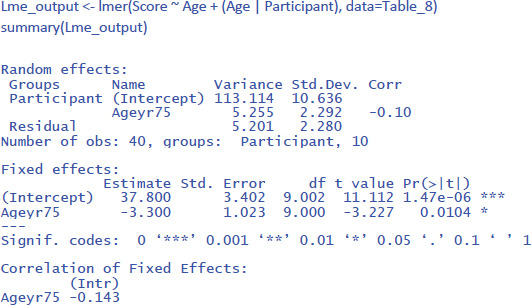

To get the value of η² in LME, we can use the package r2glmm (Jaeger, 2022), which calculates marginal R², the equivalent of η². Because the package uses an algorithm pioneered by Nakagawa and Schielzeth (2013) and extended by Johnson (2014), the method is called nsj.

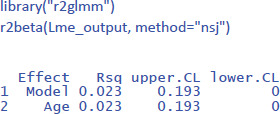

All in all, we have seen how we can analyze a repeated measures design with LME. It requires you to have more than 1 observation per condition per participant (often the case in research) and has the same advantages as we saw for the analysis of independent groups: (1) you do not need to calculate means (you can simply use all values in long format like in Table 8), (2) the model deals efficiently with missing observations, and (3) the model can be extended to more than one random variable (Part 2).

### 1.3. A design with two repeated measures

A design often includes two repeated measures, because the researchers expect an interaction between the variable they are investigating and a new variable. Such a study is done to test theories about cognitive processes underlying performance. If the theory is correct, the researchers predict a strong effect for one type of stimulus but not for another. When setting up such an experiment, it is important to make sure in advance that the interaction is informative, because often interactions do not convey much information when they are part of a design with significant main effects (Garcia-Marques et al., 2014; Wagenmakers et al., 2012). Interactions also require many more observations to be tested with the same power as main effects (a helpful rule of thumb is 4 times more; Brysbaert, 2019; for more information, see also Blake & Gangestad, 2020; Hyatt et al., 2022; Sommet et al., 2023).

Another origin of interaction effects is due to stimulus and sequence effects that researchers want to control. Interactions here can be large and crossed, but they are usually not of theoretical importance because they are about control variables (we will see in the discussion section that LMEs can reduce the number of such variables you need to include in your design).

To illustrate how to examine interaction effects, we created a toy data set with a crossed interaction and no main effects,. Participants responded to two stimulus types on two different days. Furthermore, there were two measurements for each type and (e.g., two parallel tests). Table 9 shows the data.

**Table 9:** Data of an experiment with two repeated measure variables (Day and Stimulus type) and two measurements per condition. Dependent variable is a hypothetical variable.


PARTICIPANT	DAY 1				DAY 2			
	
	STIMULUS TYPE 1		STIMULUS TYPE 2		STIMULUS TYPE 1		STIMULUS TYPE 2	
			
	MEAS1	MEAS2	MEAS1	MEAS2	MEAS1	MEAS2	MEAS1	MEAS2

Participant 1	3	3	5	5	5	3	1	4

Participant 2	5	7	4	7	4	5	4	3

Participant 3	4	3	4	4	5	6	2	3

Participant 4	1	2	4	5	6	5	3	2

Participant 5	6	4	8	8	8	9	7	9

Participant 6	5	4	5	7	7	7	5	6

Participant 7	6	6	7	7	7	4	8	5

Participant 8	4	4	5	3	6	4	5	4

Participant 9	5	3	6	3	5	6	6	7

Participant 10	7	8	9	7	7	9	8	9


#### 1.3.1. Analysis with ANOVA

**Figure 10:**
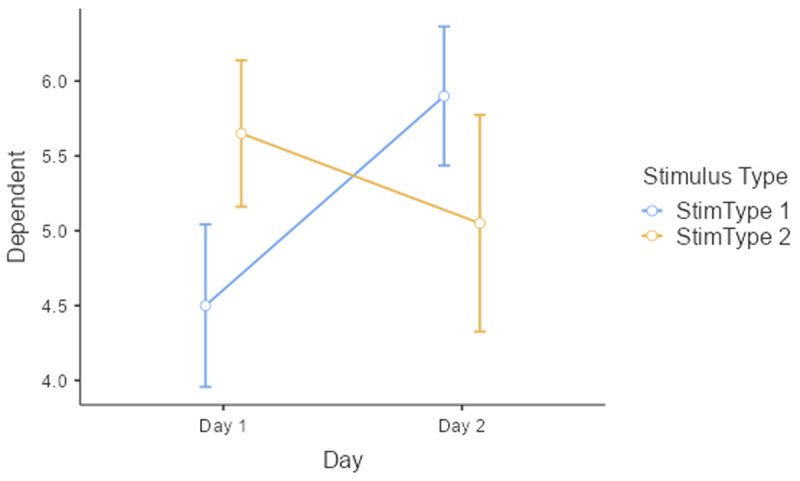
Interaction between Day and Stimulus type. The figure includes the standard errors around the means (based on jamovi).

Before starting an analysis, it is important to have a look at the data. Figure 10 shows mean performance as a function of Day and StimulusType. This shows that on Day 1 performance was higher for Stimulus type 2, whereas on Day 2 performance was better for Stimulus type 2. There was no big difference in performance between Day 1 and Day 2.

The most familiar analysis of an experimental design with several independent variables is ANOVA. For such an analysis, we have to average over measurement, because ANOVA requires a single observation per participant per condition.^13^ The outcome is shown in Table 10. This table also includes the means and the standard deviations of the conditions, showing the interaction between Day and Stimulus type.

**Table 10:** Input for jamovi and R to run a 2 × 2 ANOVA of the data of Table 10, together with the means and the standard deviations of the conditions.


PARTICIPANT	d1s1	d1s2	d2s1	d2s2

Participant 1	3	5	4	2.5

Participant 2	6	5.5	4.5	3.5

Participant 3	3.5	4	5.5	2.5

Participant 4	1.5	4.5	5.5	2.5

Participant 5	5	8	8.5	8

Participant 6	4.5	6	7	5.5

Participant 7	6	7	5.5	6.5

Participant 8	4	4	5	4.5

Participant 9	4	4.5	5.5	6.5

Participant 10	7.5	8	8	8.5

*M =*	*4.5*	*5.65*	*5.9*	*5.05*

*SD =*	*1.72*	*1.55*	*1.47*	*2.29*


To run a repeated measures analyses in R it is better to work with a long format, with 40 lines of data and extra columns for Day (Day1 vs. Day2) and StimulusType (Stim1 vs. Stim2). The code to do so can be found in the R file on osf. Once we have the right format, this is the code to run the repeated measures ANOVA in R (data from Table_10long that was made in R):

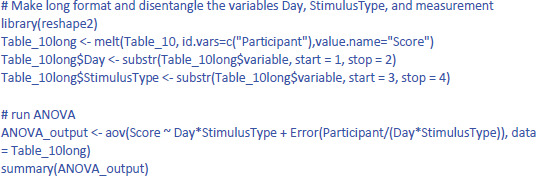

The output is:

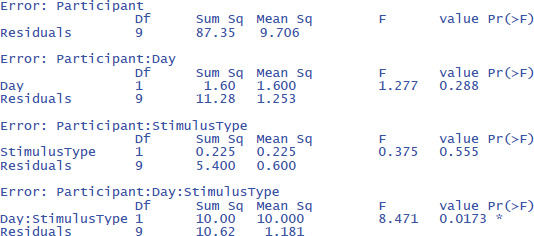

The output confirms that there are no significant main effects, but that the interaction is significant: F(1,9) = 8.471, p = .0173.

The effectsize package (Ben-Shachar et al., 2023) allows us to easily calculate eta squared (similar to d_av_) and partial eta squared (similar to d_z_) for ANOVAs. First we calculate η². Remember that this is the percentage of variance explained by each effect relative to the total variance present in the data (including the variance in overall performance between participants).

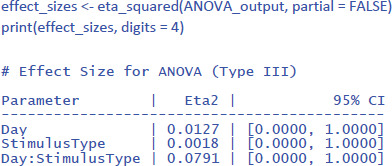

The values of η² tend to be low, because they are calculated relative to the variability in the entire dataset. This is informative in some respects (e.g., to know how much variance would be explained in a between-subjects design when differences in overall performance between participants are important as well). However, it is uninformative in other respects. Researchers use a repeated measures design precisely to disregard overall differences in performance. So, an alternative measure is one that ignores the intercepts (i.e., average performance differences) of the participants.

One would hope that partial eta squared does this, like in the design with one independent variable we saw earlier, but this is not the case. Let us first see what values we obtain, using the package effectsize:

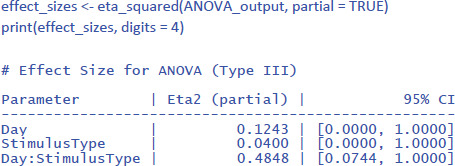

**Figure 11:**
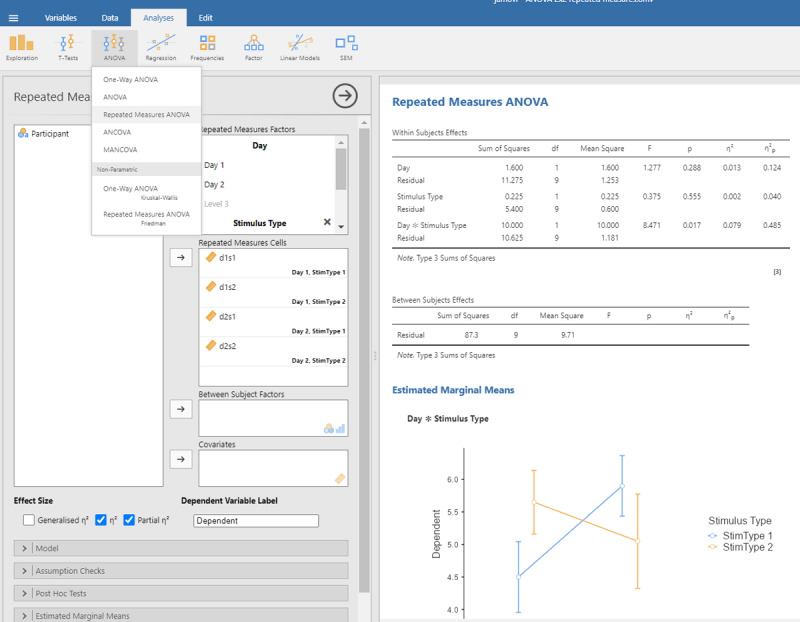
Output jamovi ANOVA 2 × 2 repeated-measures design.

Jamovi gives the same information if you check the right boxes, as can be seen in Figure 11.

We can see that the values of η²_p_ are very high in multifactorial designs with repeated measures. Whereas the interaction between Day and Stimulus Type accounted for 7.91% of the total variance, it suddenly seems to account for a whopping 48.48% of the variance that matters. Even worse, the η²_p_ indices of the main effects and the interaction effect together seem to account for 12.43 + 4.00 + 48.48 = 65% of the variance. Indeed, in many multifactorial studies with repeated measures, the sum of η²_p_ values exceeds 1 (Norouzian & Plonsky, 2018), which is impossible if η²_p_ gives information about the percentage of variance accounted for by the factors we are investigating. What is going on?

The issue with η²_p_ is that the percentage of variance is not calculated against the total within-variance, but only against the error term associated with the effect itself (i.e., after taking away all other possible sources of variance). So, η²_p_ of Day is obtained as follows:


\[
\eta_{p\_Day}^2{ = }\frac{{1.60}}{{1.60\,{ + }\,11.28}}{ = }.124
\]

The same is true for the other two effects (Stimulus Type and the interaction). The calculation of η²_p_ is correct if we want η²_p_ to convey the same information as d_z_ (or the t-value for that matter) but the measure tells us little about the importance of a variable to explain variance in the phenomenon we are examining. Even worse, an effect that explains more variance can have a lower η²_p_ than an effect that explains less variance, because the denominators are different.

Because η²_p_ does not give information about the percentage of variance accounted for, no researcher really pays attention to η²_p_ values (Norouzian & Plonsky, 2018; Richardson, 2011). The value is duly reported, because editors and reviewers ask for it, but it actually puts everyone on the wrong foot. If we look at studies investigating correlations directly, we see that the vast majority of correlations between related variables in psychology are between r = .10 and r = .40 (Bartos et al., 2024; Bosco et al., 2015; Götz et al., 2022; Lovakov & Agadullina, 2021; Schäfer & Schwarz, 2019; Sladekova et al., 2023). This corresponds to R² or η² values between .10² = .01 and .40² = .16. Still, in multifactorial experiments with repeated measures, we regularly see η²_p_ values of more than .40 (suggesting a correlation of more than r = .63 = sqrt(.40)).

Partial eta squared has been made popular by SPSS (Richardson, 2011) and seems to be added to manuscripts as part of a self-imposed ritual rather than because it informs readers.

We think it is time to correct the situation. In experimental designs like the one in the example, what we want to know from an eta squared measure is how much meaningful variance it explains, similar to what happens when we calculate η²_p_ in an ANOVA with one within-participants factor (section 1.2.1). Looking at the ANOVA table, we need to compare the variance explained by an effect to the total variance observed within subjects, disregarding the variance between participants. The total variance within subjects is obtained by totaling all six sums of squares in the within subjects panel of the ANOVA table. We call the ratio of the sum of squares due to an effect relative to the total sums of squares within participants **eta squared within (η²_w_)**.

For the example we are discussing, this gives the following values:


\[
\begin{matrix}
\eta _{w\_Day}^2\, &= \,\frac{{1.60}}{{1.60\, + \,11.28\, + \,.225\, + \,5.40\, + \,10.00\, + \,10.62}}\, &= \,.041\\
\eta _{w\_StulusType}^2 &= \frac{{.225}}{{1.60\, + \,11.28\, + \,.225\, + \,5.40\, + \,10.00\, + \,10.62}} &= .006\\
\eta _{w\_Interaction}^2\, &= \,\frac{{10.00}}{{1.60\, + \,11.28\, + \,.225\, + \,5.40\, + \,10.00\, + \,10.62}}\, &= \,.256
\end{matrix}
\]

Together, the three effects account for 30.3% of the variance within participants. The rest is noise (residual variance) in the within-participant data.

While ANOVA tables in R or jamovi make it easy to exclude between-participants variance, current implementations of LME packages lack such functionality. Fortunately, there is an alternative approach to neutralize differences between participants. It consists of centering the data per participant. We do so by subtracting the participant’s mean from their data points. The procedure is illustrated in Table 11.

**Table 11:** Table to show how to center the values of Table 10 for each participant.


PARTICIPANT	d1s1	d1s2	d2s1	d2s2	MEAN	d1s1C	d1s2C	d2s1C	d2s2C

Participant 1	3	5	4	2.5	3.625	–0.625	1.375	0.375	–1.125

Participant 2	6	5.5	4.5	3.5	4.875	1.125	0.625	–0.375	–1.375

Participant 3	3.5	4	5.5	2.5	3.875	–0.375	0.125	1.625	–1.375

Participant 4	1.5	4.5	5.5	2.5	3.5	–2	1	2	–1

Participant 5	5	8	8.5	8	7.375	–2.375	0.625	1.125	0.625

Participant 6	4.5	6	7	5.5	5.75	–1.25	0.25	1.25	0.25

Participant 7	6	7	5.5	6.5	6.25	–0.25	0.75	–0.75	0.25

Participant 8	4	4	5	4.5	4.375	–0.375	–0.375	0.625	0.125

Participant 9	4	4.5	5.5	6.5	5.125	–1.125	–0.625	0.375	1.375

Participant 10	7.5	8	8	8.5	8	–0.5	0	0	0.5


To illustrate the procedure of calculating **participant centered values**, we take observation d1s1 from Participant 1. This observation has a value of 3. The mean value of Participant 1 over the four within conditions is 3.625. Subtracting the mean from the observed value gives the participant centered value of 3 – 3.625 = –.625. The same procedure is applied to all other values in Table 11.

**Figure 12:**
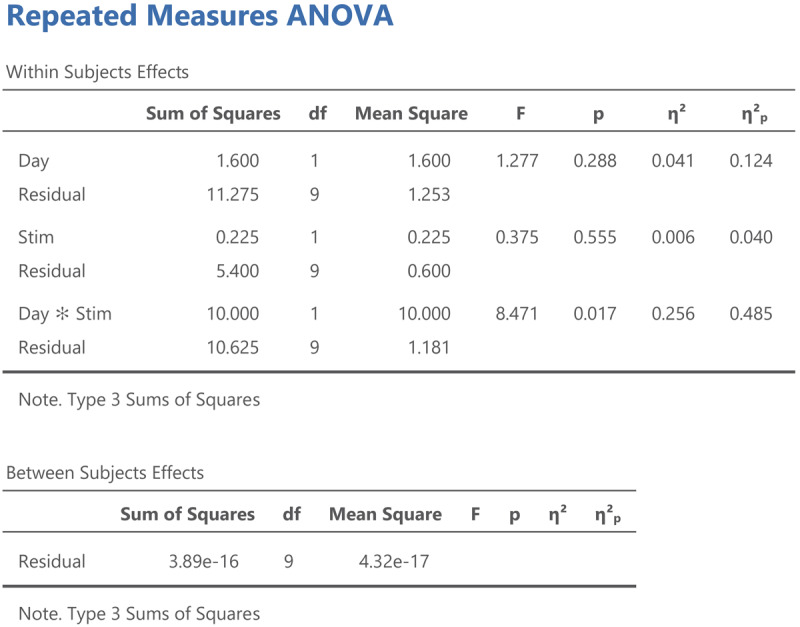
Jamovi output for an ANOVA on the participant centered values from Table 12.

Now we can look at what an ANOVA gives for participant centered values. Figure 12 shows the jamovi output.

Three things are noteworthy:

1. All the values in the Within Subjects Effects panel except η² are the same.
2. The sum of squares in the Between Subjects Effects panel is 0.^14^
3. The η² values in the Within Subjects Effects panel now match the η²_w_ values we calculated.

All information in Figure 12 is as it should be, because we reduced the sum of squares between subjects from 87.3 in the original analysis to 0 in the analysis with participant centered values, but everything else remained the same. In the next section we will see that we can use the same procedure to get the η²_w_ output from LME models.

When we have a significant interaction, we are likely to calculate the effect sizes of the most interesting pairs of conditions with the equations we have discussed under 1.2 (paired conditions). So, for the present study, we may want to test how large the difference between the Stimulus types is on Day 1 and on Day 2.

#### 1.3.2. Analysis with LME

To run an LME analysis we need Table 9 in long format. This table will have 80 rows (10 participants * 2 days * 2 stimulus types * 2 measurements) and five columns (Participant, Day, StimulusType, Measurement, Score).

For the LME analysis, we have three fixed effects: the main effect of day, the main effect of stimulus type, and the interaction. In addition, we have four random effects: intercept per participant, slope of day per participant, slope of stimulus type per participant, and slope of the interaction effect per participant (remember that we need random slopes for all within effects).

**Figure 13:**
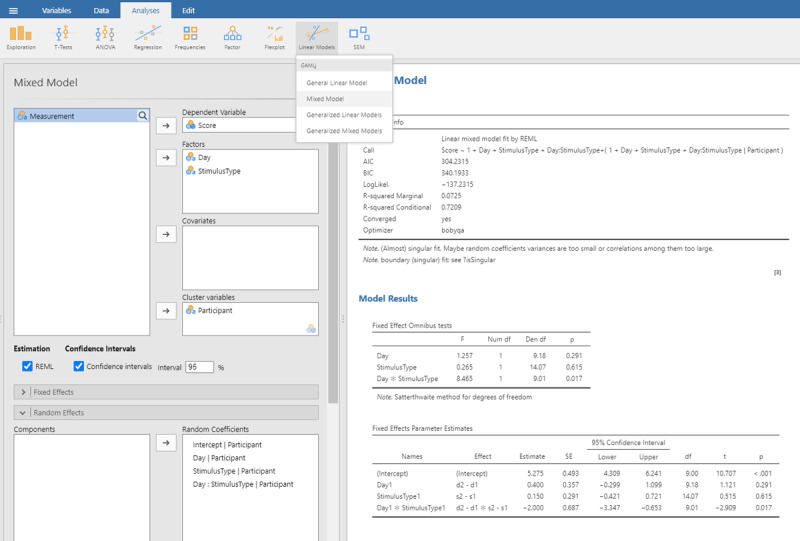
Output jamovi LME analysis 2 × 2 repeated-measures design.

We start with the jamovi output (Figure 13), because it is simpler.

For better visibility, we repeat the table showing the estimates of the fixed effect parameters.

**Table:** 

Fixed Effects Parameter Estimates						

**NAMES**	**EFFECT**	**ESTIMATE**	**SE**	**df**	**t**	**p**

(Intercept)	(Intercept)	5.275	0.493	9.00	10.707	<.001

Day1	d2 – d1	0.400	0.357	9.18	1.121	0.291

StimulusType1	s2 – s1	0.150	0.291	14.07	0.515	0.615

Day1 * StimulusType1	d2 – d1 * s2 – s1	–2.000	0.687	9.01	–2.909	0.017


We can easily verify that the statistics of the fixed effects are very similar to what we obtained in the ANOVA and t-tests on the averaged data.

In addition, we get information about the random effects.

**Table:** 

Random Components				

**GROUPS**	**NAME**	**SD**	**VARIANCE**	**ICC**

Participant	(Intercept)	1.511	2.283	0.665

	Day1	0.835	0.697	

	Stimulus type1	0.522	0.273	

	Day1 * Stimulus type1	1.556	2.421	

Residual		1.073	1.152	

Note. Number of Obs: 80, groups: Participant 10				


The table with random effects tells us that in our toy example all random intercepts have variance associated with them, but that the variability of Stimulus type is rather small.^15^

Finally, we are informed that the fixed effects together explain 7.2% of the variance (R² marginal = .0725).

We discussed the jamovi output first, because lme4 and lmerTest have an unpleasant surprise in store for the uninformed user. The commands one would expect for the LME test are:

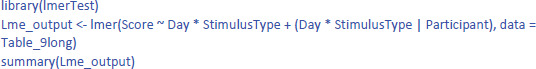

This gives the following output:

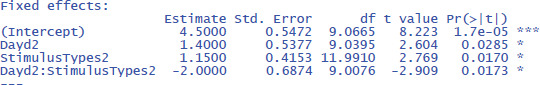

As you can see, the output of these commands does not at all correspond to what we have seen so far and what we would expect on the basis of the ANOVA! All effects seem to be significant, also the main effects, even though it is clear from Figure 10 that there are no differences between Day 1 and Day 2, or between Stimulus type 1 and Stimulus type 2 (as we also saw in the ANOVA analysis). Only the p-value for the interaction is the same. What is going on here?

The above analysis is an illustration of the observation that software packages sometimes include default choices that deviate from users’ intuitions (Brehm & Alday, 2022). As we saw before, the packages lme4 and lmerTest use the default dummy coded contrasts (0 and 1) for categorical variables. In the case of two levels, one level is chosen as the 0 value (in our example, these are Day 1 and Stimulus type 1) and the other as 1 (Day 2 and Stimulus type 2). What the programs then do is calculate whether there is a main effect in the 0 reference condition (i.e., is there a main effect of Day for Stimulus type 1, and is there a main effect for Stimulus type at Day 1?). As you can see in Figure 10, these specific main effects are indeed present, but they are absorbed by the wider interaction.

Dummy coded contrasts is relevant in regression-like situations, but for designs with orthogonal factors in experimental research, they must be avoided. In order for the analysis to correctly answer our research questions, the reference point must be in the middle of Figure 10: between Day 1 and Day 2, and between Stimulus type 1 and Stimulus type 2. This is achieved by sum contrast coding. So, we can try the following code:

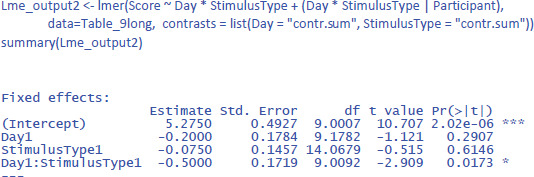

This solves the issue of the t-values and p-values, but halves the regression weights of the main effects, because the default contrast weights in lme4 (and R in general) are –1 and +1. For the interaction, the effect estimate is even only one quarter of the difference in the data. Therefore, the jamovi gamlj option is more convenient. 16 You get it in R with following code:

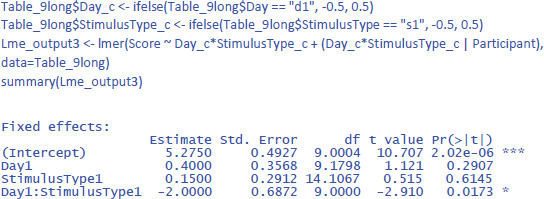

Now the effects match what we expect.

Package r2glmm can be used to get the values of η².

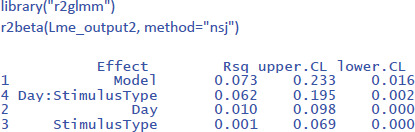

To get the values of η²_w_, we need to work with participant centered scores. You can calculate these values with Excel as we did in Table 11, but you can also use the following command in R to calculate participant centered scores in the long format table:

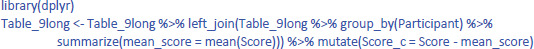

For the LME analysis with centered scores, one would think that it makes sense to take out the participant intercept as random variable, because there are no differences between participants anymore. However, current implementation of lme4 will then calculate separate random effects for each level of the centered variable, which may be even worse. So, better to leave the intercept in the model and ignore it. Having the intercept in the model does not make any difference in the calculation of η²_w_, because that value is determined relative to the total variance, independent how it is allocated over components. So, the only thing that must be changed in the model is the dependent variable, which is the centered score instead of the original score:

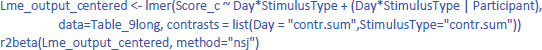

This gives us the following values of η²_w_:

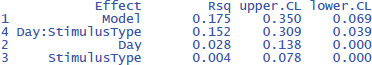

The η²_w_ values of the LME analysis are lower than those of the ANOVA analysis (together explaining 17.5% of the within variance instead of 30.3%). This is because in the ANOVA analysis we eliminated within-subjects variance by taking the mean of two measurements. The η²_w_ values of the LME analysis are more informative because they remain the same regardless of the number of observations per participant per condition, while those of the ANOVA analysis typically increase as more observations are averaged. As a result, the standardized effect sizes based on ANOVA analysis may differ considerably between studies (Brysbaert & Stevens, 2018).^17^

While examining the various standardized effect sizes that have been proposed, we encountered a further problem with the η²_p_ values, as currently calculated by the R package effectsize (and probably by other packages as well). The issue is as follows. The effectsize package calculates η²_p_ based on the F-values in an ANOVA table. This is valid for ANOVA and simple regression, but not always for LME, because the degrees of freedom in LME are not well defined. This became clear when we calculated η²_p_ for the current example. The η²_p_ estimate of the interaction was very different if it was based on a model with random Day * StimulusType slopes (effect size is η²_p_ = .48) than if it was based on a model without random Day * StimulusType slopes (same effect size is η²_p_ = .20), even though the two models did not differ significantly in how well they fitted the data (see the accompanying R code for more information). Note also the unrealistically high estimate of the effect size for the interaction. A value of η²_w_ = .152 tells us that 15.2% of the variance within participants is explained by the interaction (equivalent to a correlation of r = .39), while a value of η²_p_ = .48 falsely suggests that the effect size would be similar to a correlation of r = .69. This is a further reason why researchers should not use η²_p_ values as estimates of effect sizes.

A second take-home message for LME analyses with interactions is to remember that you must make sure that the independent variables are centered on the mean (sum coding). This also applies to regression analysis if the analysis includes interactions between predictors. Otherwise, you are likely to misinterpret the outcome of your statistical analysis!

It is wise always to make a plot of your data, so that you can check whether the outcome of the analysis aligns with the data. Adding a plot in gamlj/jamovi is easy, as you only have to check a box to get a figure added. For a 2 × 2 design, you can use the package CGPfunctions. This is the code to use to get a picture like Figure 10.

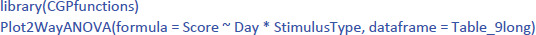

## 2. Adding stimuli as a second random variable to LME analysis

So far we have discussed LME analysis as an extension of t-tests and ANOVAs, where we have seen that it is an interesting alternative when you have collected several observations per condition, because (1) you can work directly with the data you have collected (you don’t need to average), (2) missing data pose fewer problems, and (3) the standardized effect sizes apply to data in the real world and not to embellished data you have created by averaging observations.

However, the real advantage of LME over simple tests becomes clear when we run studies in which we not only want to generalize to new participants but also to other stimuli. In the previous examples we had two measurements per condition, but these measurements were repetitions of the same experience. We did not expect any differences between them and did not want to extend our conclusions to other measurements. However, for many research questions in psychology we want to make claims that are true not only for all people of the population tested but also for all stimuli of the kinds we presented (Brysbaert & Stevens, 2018; Judd et al., 2012; Lewis, 2023; Yarkoni, 2022). The stimuli we presented were only a random sample of the stimuli we could have presented and for which we want to make conclusions.

### 2.1. Why stimulus generalization is important

Suppose you want to test the idea that people value individuals from their own group more than individuals from another group. To investigate this, you show pictures of faces of 18-year-olds to a group of 18-year-olds and to a group of 75-year-olds. You ask the participants to rate the attractiveness of the faces on a scale from 1 (not attractive) to 7 (very attractive). You predict that the average rating of the 18-year-old participants will be higher than that of the 75-year-old participants. Further suppose that you test 10 participants in each group and that you present them with 10 faces. Table 12 shows the data you obtain.

**Table 12:** Data from the study of face attractiveness (S1 = stimulus 1, P1 = participant 1, yr18 = 18-year-old, yr75 = 75-year-old). Dependent variable is attractiveness rating on a Likert scale from 1 (unattractive) to 7 (attractive).


PARTICIPANT	AGE	S1	S2	S3	S4	S5	S6	S7	S8	S9	S10

P1	yr18	4	3	2	4	2	5	3	5	1	6

P2	yr18	5	4	5	5	4	5	3	5	4	5

P3	yr18	5	3	2	5	2	2	5	5	2	3

P4	yr18	4	3	3	3	1	1	4	5	4	5

P5	yr18	7	2	4	6	2	3	3	4	2	4

P6	yr18	7	5	1	6	2	5	5	5	3	7

P7	yr18	7	3	2	4	1	4	3	5	5	5

P8	yr18	5	4	4	3	3	1	3	4	4	6

P9	yr18	4	4	5	4	2	5	4	5	4	5

P10	yr18	6	3	3	5	2	2	4	5	4	7

P11	yr75	6	2	5	3	1	4	1	3	3	5

P12	yr75	6	1	2	5	3	4	4	1	1	4

P13	yr75	5	2	1	2	3	2	3	1	1	5

P14	yr75	4	3	4	5	2	5	5	3	3	7

P15	yr75	4	1	4	3	1	3	1	1	1	5

P16	yr75	6	3	4	6	2	4	4	1	2	3

P17	yr75	7	3	5	6	4	4	2	2	2	4

P18	yr75	7	5	5	2	2	5	4	4	3	5

P19	yr75	5	3	5	4	2	3	2	1	3	5

P20	yr75	6	1	1	5	2	3	1	1	3	4


If we calculate the mean rating per participant over the 10 stimuli, we see that the ratings of the 18-year-olds (M = 3.89, SD = .45) on average are higher than those of the 75-year olds (M = 3.30, SD = .64). We can use a t-test or ANOVA for independent samples, to see whether this difference is statistically significant. The t-test informs us that the difference is significant at the .05 level: t(18) = 2.38, p = .029, d = 1.06. The ANOVA additionally tells us that η² = η_p_² = .239 (there is no value of η_w_² because Age is a between-participants variable). So, do we find evidence for our hypothesis?

**Figure 14:**
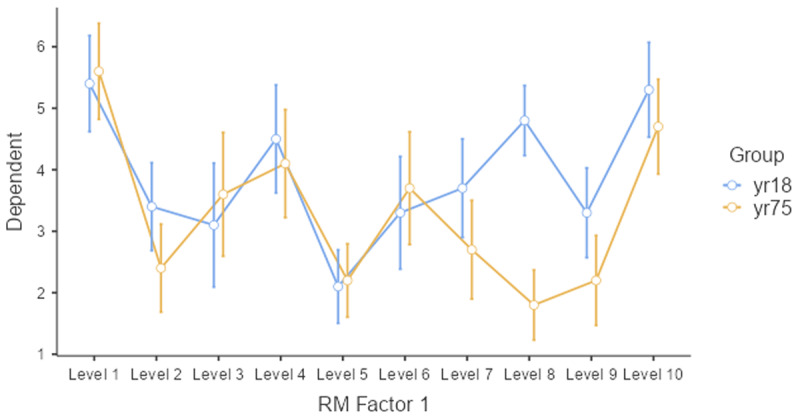
Average ratings of the young and old group per stimulus.

The evidence is less convincing when we look at the average rating per face stimulus. This is shown in Figure 14.

Only two or three stimuli show a higher rating for the 18-year-olds, and most of the difference is due to stimulus 8. So, what looks like an age effect, could very well be a stimulus effect. The young participants liked face 8 much more than the old participants. One way to test whether the effect is caused by a small subset of stimuli, is to run a paired-samples t-test or ANOVA by stimuli. If the age difference is seen for most of the stimuli, the Group effect will be significant. Otherwise, it will not be significant. Table 13 shows what this data looks like.

**Table 13:** Summary table for a related-samples t-test across stimuli.


STIMULUS	yr18	yr75

S1	5.4	5.6

S2	3.4	2.4

S3	3.1	3.6

S4	4.5	4.1

S5	2.1	2.2

S6	3.3	3.7

S7	3.7	2.7

S8	4.8	1.8

S9	3.3	2.2

S10	5.3	4.7


According to this analysis, the difference between the young and the old participants is not significant: t(9) = 1.8, p = .106, two-tailed, d_z_ = .568, η² = .067, η_p_² = η²_w_ = .264. Notice that in this analysis the observations are paired, because each picture was rated both by the young and the old participants. So, we can calculate η²_w_; the value is the same as η²_p_, because there is only one independent variable and one observation per cell.

So, what we have in this study is a difference between two groups of participants that could well be due to one or two stimuli and not to the fact that 18-year-olds rate faces of 18-year-olds as more attractive than 75-year-olds because the faces belong to their in-group. With a different set of faces, the results could look different (Clark, 1973; Yarkoni, 2022).

There are two solutions to the issue of stimulus generalization. First, researchers can do what we did above: run a t-test by participants and a t-test by stimuli and conclude that there is a genuine in-group effect *only when the difference is significant in both analyses*. This is the type of analysis language researchers have done for years (other research fields have been less aware of the issue of stimulus generalization). The first analysis is typically called F1 analysis (by participants); the second F2 analysis (by stimuli). A better alternative, however, is to run a LME analysis with both participants and stimuli as random variables (Baayen et al., 2008; Brysbaert & Stevens, 2018; Judd et al., 2012; Lewis, 2023; Yarkoni, 2022).

### 2.2. LME analysis of the face rating study (one independent variable that is a between-groups variable by participants and a repeated measure by stimuli)

If we want to generalize over participants and stimuli, we must indicate that both participants and stimuli are random variables. This means that we assign an intercept to each participant and to each stimulus. In addition, because the observations are paired across stimuli, we must enter a random slope for stimuli (as discussed in sections 1.2.1 and 1.2.2).

To run the analysis we need to enter the data in long format. The table will contain 200 data lines (20 participants * 10 stimuli) and four columns (Participant, Age, Stimulus, Rating). You can do so in Excel or using the following R code:

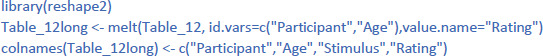

#### 2.2.1. Separate analysis by participants and by stimuli with LME

First, it may be interesting to remember that we can use LME analyses as an alternative to the t-tests by participants and stimuli described in 2.1. All we need to do is to run one analysis with participants as random variable and one analysis with stimuli as random variable.

For the analysis by participants (between-subjects variable, so no random slopes), this gives the following output (compare with the t-tests above):

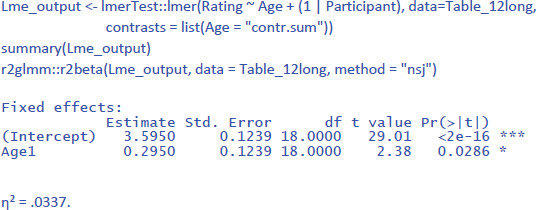

Notice that η² is much lower than in the F1 ANOVA (where η² = .239). This is because in the ANOVA analysis we averaged over the 10 stimuli. The value of η² in LME gives the percentage of variance accounted for by Age in the ratings to the pictures. The value of η² in the ANOVA gives the percentage of variance accounted for by Age in average ratings over 10 pictures.

To some extent, η² is an underestimate of the effect size, because the same pictures were given to both groups of participants and we are not interested in differences between the pictures. So, it makes sense to calculate η²_w_ with stimulus centered scores. This is done as follows:

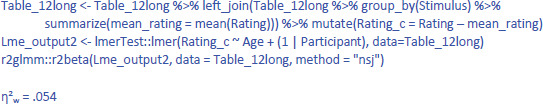

As expected, the value of η²_w_ is in-between the η² values obtained in the LME analysis and in the ANOVA. If we calculate η²_p_ with the package effect size, we get the same η²_p_ as for the ANOVA (η²_p_ = .239). It is an indication of the significant t-value obtained, but does not inform us about the size of the Age effect.

For the analysis by stimuli (repeated measure, so both random intercepts and random slopes), the LME analysis looks as follows:

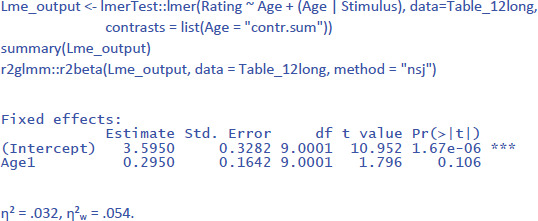

Because the data set is the same in the analysis by stimuli as in the analysis by participants, the values of η² and η²_w_ are very much the same. For comparison, η² and η²_p_ in the F2 ANOVA were .067 and .264.

#### 2.2.2. Combined LME analysis with both participants and stimuli as random variables

The analyses by participants and stimuli were interesting, because we could compare them with the F1 and F2 ANOVAs. However, a strength of LME is that it allows you to run combined analyses over both participants and stimuli, allowing you to generalize across both. If only participants are considered as random variable (and data are averaged across stimuli), we run the risk of drawing conclusions that are valid only for the specific stimuli used. If only stimuli are considered as random variable (and data are averaged across participants), we run the risk of drawing conclusions that are valid only for the specific participant group tested. In addition, the effect sizes will be exaggerated because they only apply to the average scores and not to the dependent variable itself.

As it happens, an LME analysis with both participants and stimuli as random variables is not more difficult to run than an LME analysis with one random variable. For the study of face ratings we have been analyzing, the following commands suffice:

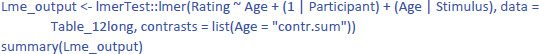

(1 | Participant) indicates that we only include a random intercept for participants (between groups variable). (Age | Stimulus) indicates that we include both random intercepts and slopes for stimuli (repeated measure). This gives us the following output:

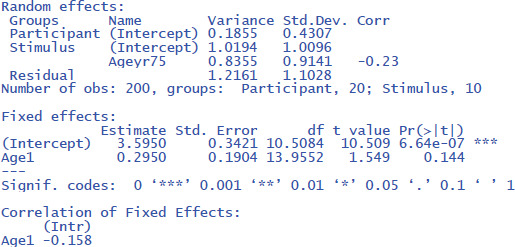

The output tells us that the effect of Age is not significant (t(14) = 1.549, p = .144). The evidence is not strong enough to assume that the effect generalizes to both new participants and new stimuli. We further see that there are three random effects (intercept of participant, intercept of stimulus, and variation of Age slope across stimuli), in addition to residual variance (the remaining variability in the data).

**Figure 15:**
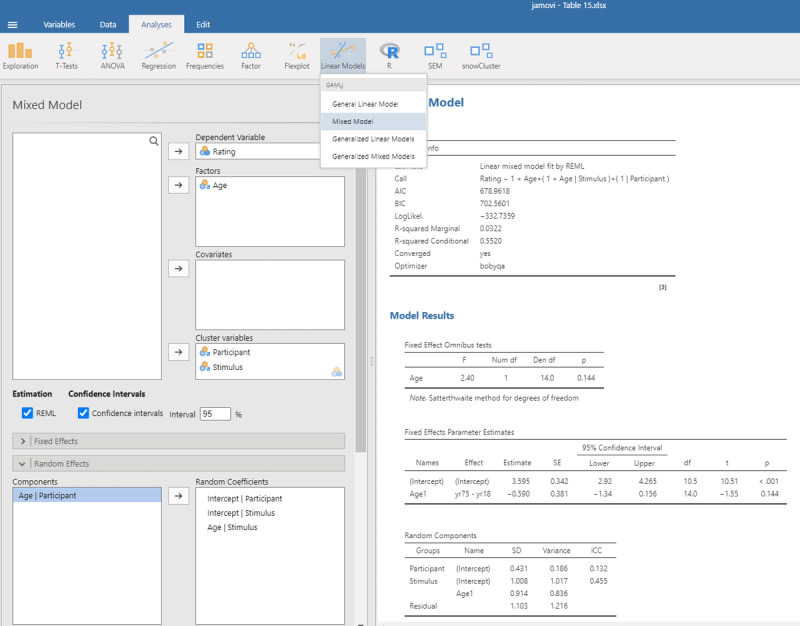
Output jamovi LME analysis face rating study.

The same outcome is obtained with gamlj/jamovi (Figure 15), which has the advantage that the interface is more intuitive and that it makes convenient default choices (e.g., the use of –.5 and +.5 coding, which does not halve the difference between the means):

The package r2glmm informs us that η² = .032 and that η²_w_ = .053. These values agree quite well with the analysis by stimuli, because there were no large differences between participants.

### 2.3. Analysis of dataset with two independent variables (repeated measures by participants, between-group variables by items)

In the final example, we introduce an interaction. Suppose a researcher wants to investigate the joint effects of language dominance and background knowledge on text reading time. Bilingual participants are asked to read texts in their native language (L1) and in their second language (L2). Half of the texts are about topics the reader is familiar with (e.g., student life), half about topics they know little about (e.g., life in 16^th^ century Korea). The researcher expects costs of L2 reading and low background knowledge. The main question is whether both variables interact.

**Figure 16:**
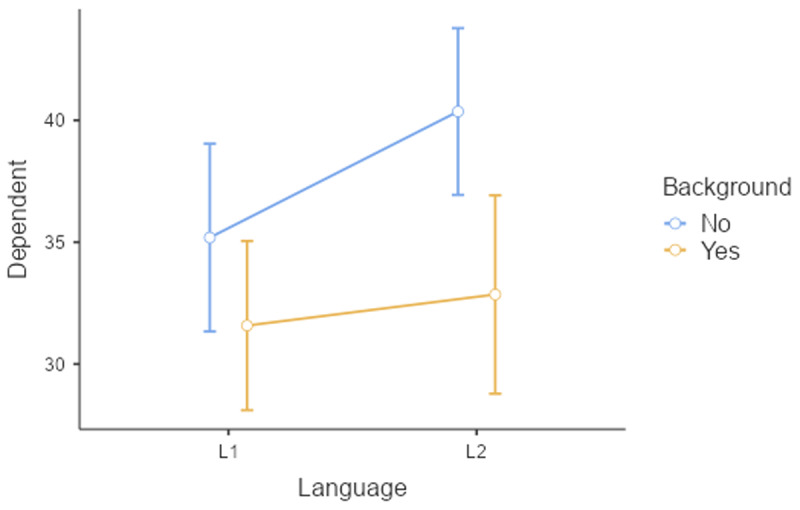
Figure of reading times as a function of Language and Background knowledge.

Table 14 gives the toy dataset we will work with. Twelve participants read 20 texts; 5 in each condition. The texts consisted of 125 words and were matched on language difficulty (e.g., frequency of the words used, length of the sentences, …). Figure 16 shows the reading time as a function of Language and Background knowledge.

**Table 14:** Data from a study on text reading (language = language of the text, background = whether or not the reader is expected to be familiar with the topic of the text, p1 = participant 1, t1 = text 1). Dependent variable is seconds needed to read a 125 word text.


LANGUAGE	BACKGROUND	TEXT	p1	p2	p3	p4	p5	p6	p7	p8	p9	p10	p11	p12

L1	Yes	t1	36	31	25	41	25	21	37	31	28	22	30	25

L1	Yes	t2	38	29	32	30	30	37	38	34	36	25	30	32

L1	Yes	t3	38	25	32	46	25	30	42	30	41	22	28	36

L1	Yes	t4	48	24	28	40	28	28	40	26	36	19	40	30

L1	Yes	t5	38	22	28	32	30	35	37	31	38	21	33	24

L1	No	t6	39	36	40	42	35	30	55	34	34	28	48	42

L1	No	t7	34	17	35	34	25	19	43	29	30	19	39	43

L1	No	t8	42	26	36	40	31	31	44	36	31	34	43	30

L1	No	t9	42	26	35	32	34	25	42	31	39	31	36	40

L1	No	t10	45	34	36	41	35	28	52	36	40	28	33	34

L2	Yes	t11	34	21	26	30	33	29	39	30	34	30	28	29

L2	Yes	t12	39	24	26	37	27	29	46	31	47	27	33	34

L2	Yes	t13	41	28	25	39	28	35	38	32	43	28	35	23

L2	Yes	t14	32	27	32	44	20	25	42	27	44	18	39	33

L2	Yes	t15	46	22	31	42	33	31	40	34	58	33	32	29

L2	No	t16	41	27	44	34	38	42	49	45	42	43	51	37

L2	No	t17	50	39	42	35	34	39	49	42	37	33	43	37

L2	No	t18	57	37	50	40	46	49	45	38	45	38	45	36

L2	No	t19	46	32	38	31	37	36	56	33	40	34	38	26

L2	No	t20	51	36	40	35	40	38	53	27	42	37	40	35


To keep the tutorial focused, we move straight to the combined analysis of participants and texts. Table 15 summarizes the main findings of the F1 and F2 ANOVAs.

**Table 15:** Findings in the analyses by participants and by texts, limited to the ANOVAs.


ANOVA BY PARTICIPANTS (F1 ANALYSIS)	ANOVA BY TEXTS (F2 ANALYSIS)

2 × 2 analysis with repeated measures	2 × 2 analysis with between-text variables

Main effect Language: F(1,11) = 13.85, p = .003, η² = .061, η²_p_ = .557	Main effect Language: F(1,16) = 9.45, p = .007, η² = .166, η²_p_ = .371

Main effect Background: F(1,11) = 22.00, p < .001, η² = .181, η²_p_ = .667	Main effect Background: F(1,16) = 28.09, p < .001, η² = .493, η²_p_ = .637

Interaction: F(1,11) = 5.66, p = .037, η² = .022, η²_p_ = .340	Interaction: F(1,16) = 3.45, p = .082, η² = .061, η²_p_ = .177


Remember that in the F1 analysis by participants we can generalize over participants, but the effect could in principle be due to a single text. Similarly, in the F2 analysis by items we can generalize over texts, but the effect could in principle be due to a single participant. To know whether our evidence is strong enough to assume that it generalizes both to other participants and other texts, we must run an LME with both participants and texts as random variables. The effects of language and background are repeated measures by participants (hence, they require random slopes) and between variables by texts (so, only random intercepts). We must use sum coding to get correct statistics for the main effects.

This brings us to the following commands:^18^

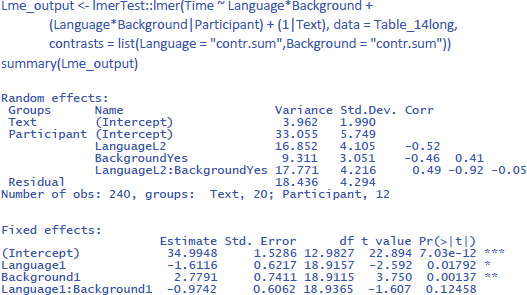

Interestingly, taking all variance into account, the findings of the study are less compelling than suggested by the separate F1 and F2 analyses. The study suggests that the main effects of the independent variables are likely to generalize to other participants and stimuli (p < .05). However, the interaction effect of interest, while consistent with the researcher’s hypothesis, does not reach statistical significance (p = .125). To get a better idea of its existence, the researcher is recommended to conduct a larger study involving a larger sample of participants and/or a broader range of texts.

Further interesting is to know how large the effects are in terms of variance accounted for. For this, we use η² and η²_w_.

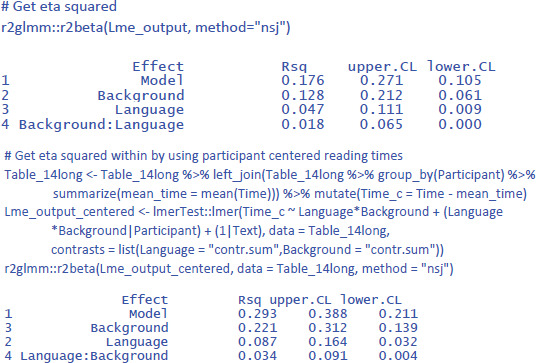

We see that Background knowledge had the largest effect, accounting for 12.8% of the total variance and 22.1% of the variance if intercept differences between participants are discarded. Language explained 4.7% of total variance and 8.7% of within-participant variance. Finally, the percentages explained by the interactions are the smallest: 1.8% of total variance and 3.4% of within-participant variance.

## 3. Discussion

This article aimed to demystify linear mixed-effects (LME) analyses in the context of pairwise comparison designs. We began by establishing the connection between LME analysis and conventional approaches like ANOVA and t-tests, when participant variability constitutes the only random effect. This initial comparison served to highlight key aspects of LME, often obscured in models with multiple random variables. Subsequently, we delved into LME analyses for designs incorporating both participant and stimulus variability. We clarified the scenarios where this approach is advantageous and offered a guide to its implementation.

We further emphasized the importance of extracting and reporting standardized effect sizes alongside condition means and standard deviations. Such effect sizes provide a measure of the magnitude of an effect relative to data variability, facilitating interpretation and comparison across studies. They are also indispensable for inclusion in meta-analyses, a cornerstone of evidence-based practice. Unfortunately, many current LME reports lack the necessary detail for extracting these standardized measures.

Similar to previous researchers, we identified limitations in using partial eta squared (η²_p_) and, to a lesser extent, eta squared (η²) for interpreting multifactorial designs with repeated measures, which constitute the majority of experimental designs in behavioral studies. The existing measures fail to address the critical question of how much meaningful variance is explained by individual factors.^19^ To solve the problem, we introduced eta squared within (η²_w_), which can also be used with ANOVA. These are the uses of the three different standardized measures of effect size:

- Eta squared (η²): analogous to Cohen’s d in pairwise comparison of independent conditions, and d_av_ in pairwise comparison of a repeated measure; it represents the percentage of the total variance explained by an effect.
- Partial eta squared (η²_p_): useful for designs with one within-subjects variable and a single observation per cell. In a pairwise comparison of two related observations with a single observation per cell, it conveys the same information as Cohen’s d_z_. Otherwise, it indicates the percentage of variance explained by an effect, when all other effects are controlled for. It a mathematical rewriting of the t- or F-value and more informative about these values than about the degree to which an effect influences the dependent variable.
- Eta squared within (η²_w_): This is a new standardized effect size proposed for related conditions. It gives the percentage of within-variance explained by an effect. The within-variance is obtained by using centered values, so that no between-variance remains.

We described how the three measures can be obtained with existing ANOVA and LME software packages by using centered values of the dependent variable.^20^ While our approach is useful, we acknowledge the possibility of alternative, more elegant solutions that are mathematically equivalent. Partitioning the random variance into within and between components, similar to what happens in traditional ANOVAs, seems a promising avenue for integration within existing software packages. We hope that more qualified colleagues will bring the concept to fruition and make η²_w_ indices as readily available in statistical packages as η²_p_ is now.

It is important that researchers learn to expect that most η²_w_ values will be smaller than .10. This corresponds to correlations smaller than r = \[
\sqrt {.10}
\] = .32. Larger standardized effect sizes are rare in psychology (Bartos et al., 2024; Bosco et al., 2015; Götz et al., 2022; Lovakov & Agadullina, 2021; Schäfer & Schwarz, 2019; Sladekova et al., 2023), also in experimental psychology when effect sizes are calculated with LME on the raw data rather than with ANOVA on data averaged over many trials (Brysbaert & Stevens, 2018). It is crucial to understand that the larger values of partial eta squared are a product of their specific calculation and are not informative as standardized effect sizes outside the statistical model used (they are not an estimate of the variance in effect slopes observed between participants).

We recognize that this article is likely to disappoint readers looking for guidance on more complicated designs. At the same time, 2 × 2 designs and pairwise comparisons are at the heart of research, and we advise readers to keep their designs as simple as possible, as these usually yield clearer interpretations and stronger conclusions. Furthermore, the examples presented here hopefully provide a solid foundation for analyzing more complex designs.

LME analyses are good for simplifying analyses for two reasons. First, the design effortlessly includes multiple observations per condition. This is different from ANOVA where observations must be averaged or independent variables added to make the number of conditions equal to the amount of data. The second reason is that intercepts for participants and stimuli capture a lot of measurement noise. In ANOVAs, a slow person or difficult stimulus can add a lot of noise in a counterbalanced design because the condition in which the person or stimulus occurs is slower than the other conditions, independent of the influence of the factors being examined. The only way to control for this noise in an ANOVA is to add a factor encoding the Latin-square group (Pollatsek & Well, 1995), which adds complexity to the design. In LME, performance differences between participants and stimuli are largely captured by differences in intercepts (a random variable), so researchers are better able to restrict their fixed effects to the variables of interest.

Returning to standardized effect sizes, we recommend that readers always give the values of η² from LME (and ANOVA) analyses. When at least one variable is a repeated measure (by participants or by stimuli), it is necessary to also give the values of η²_w_. If you provide access to the η² and η²_w_ values, readers can better understand the value of your finding relative to other findings and include your findings in meta-analyses. The values can be added to tables, as in Figure 12, or they can be added in the main text when the effect is described. So, for the interaction effect between language and background knowledge in the reading study of Section 2.3, we could write something like: “There was no significant interaction between Language and Background knowledge in an LME analysis with participants and texts as random variables (t(18.94) = –1.607, p = .125, two-tailed, η² = .018, η²_w_ = .034).” We also recommend that researchers make their data and analysis code accessible using an online repository like OSF.

## 4. Further reading

The present article provides an overview of basic experimental designs. Readers seeking more in-depth coverage of more complex designs (including those involving continuous predictors) may refer to Brauer and Curtin (2018), who also provide a succinct summary of the material covered here. For additional examples, Brown (2020) also offers a valuable resource. Meteyard and Davies (2020) review best practices of LME models in psychological science.

## Footnotes

Another solution is to make the model more complex by using two variables: Age (between groups) and Day of testing (repeated measure). Then there is only one observation per cell of the design. This complicates the design, especially if no effect of day is expected. Below we will see how LME provides a third, simpler solution.

All p-values in this tutorial are two-tailed, as this is the default output of most software programs. We assume that there is no information in advance about the direction of the difference between the two conditions.

The easiest way to do this is to work with RStudio. There are many websites and videos on the Internet explaining how to import an Excel file in RStudio.

Because the Welch test does not assume equal variances in the groups.

If you already activated the package effectsize, you can drop the first line. You can also calculate the value of η² by hand. Simply divide the Sum of Squares of the effect by the total Sums of Squares in the ANOVA table. So, η² = 125/(125 + 2181) = .054.

This a good precaution, because some beginning students assign numbers to categorical conditions (e.d. 1 = condition 1 and 2 = condition 2). Many R programs automatically interpret such variables as continuous variables and will not calculate what you think they should calculate.

The numbers you obtain may differ slightly from those reported here because LME works with random number generation and the numbers generated in your analysis are likely to differ from those generated in the analyses here. However, the differences will be small and should not lead to any conclusions other than those reported here.

It is also possible to calculate a d-effect size for this simple LME model (Westfall et al., 2014; see also Brysbaert & Stevens, 2018), but that effect size does not generalize easily to more complex LME designs. Therefore, we do not discuss it here.

An even better way to help readers understand your data is to make it available along with the analysis code in a robust repository, with a link in the manuscript.

The values of η² and η²_p_ overestimate the true effect size for small samples. This is a good reason not to conduct repeated measures designs with fewer than 50 participants and between-groups designs with fewer than 100 participants per group (see also Brysbaert, 2019).

The ≈ sign indicates that the value is slightly overestimated because df = N – 1. The larger the sample, the closer the approximation (Brysbaert, 2019). If desired, the equation can be extended to take this factor into account.

You also see an ICC number. This number in gamlj is not very useful. It says how much of the variance is explained by the intercept differences between the participants. ICC = interceptpart/(interceptpart + residual variance) = 112.09/(112.09 + 5.20) = .956, which does not tell us anything about the test.

Again, an alternative often used in the past was to add an extra variable with two levels (Measurement), making ANOVAs extra complicated.

For all practical purposes, a value of 3.89e–26 (or = 0.000000000000000389) can be equated to 0, although mathematically there are infinitely many numbers between 3.89e–16 and 0.

ICC is computed as = intercept_part_/(intercept_part_ + residual) = 2.283/(2.283 + 1.152) = .665, which is not informative for the calculation of effect sizes.

The contrast coding in gamlj works well for designs with variables that have two levels, but may need extra scrutiny for variables with more levels, because the default contrast weights in R were chosen so that they generalize to any number of levels and variables. The quality of the default choices in gamlj can be tested by comparing them with the factor coding option “deviation”. This gives the same outcome of the sum contrast coding option in R. Alternatively, the quality can also be tested by comparing various commands in R.

In the R code related to section 1.2.2 you can see that there also was a difference between η²_w_ and η²_p_ in the LME analysis of the longitudinal study of working memory capacity with Age as within-participants variable. Whereas η²_p_ for the LME analysis was .54 (like in the ANOVA), η²_w_ decreased to .345, in line with the fact that the variance of raw scores is larger than the variance of averaged scores (SD = SE * sqrt(N)).

Readers who run the code will notice that the analysis sometimes gives a boundary (singular) fit warning, because the model does not converge well. This is a wider issue, certainly when one uses small datasets. More information about convergence issues in LME is given by Matuschek et al. (2017) and Park et al. (2020).

Olejnik and Algina (2003) and Bakeman (2005) proposed another solution for multifactorial designs, which they called generalized eta squared (η²_G_). This solution consists of excluding the sum of squares of the other factors from the denominator of eta squared. So, for the effect of Day in the 2 × 2 study described in Table 10, the sum of squares of the effects of StimulusType (0.225) and Day * StimulusType (10.000) are excluded from the denominator. However, the between-subjects sum of squares (87.3) is retained, just like in eta squared but unlike eta squared within. For repeated measures designs with small effect sizes and large individual differences in overall performance, generalized eta squared will resemble eta squared very much. For instance, for the study described in Table 10, η²_G_Day_ = .014, η²_G_StimulusType_ = .002, and η²_G_Interaction_ = .080, very similar to η² and considerably lower than η²_w_. Generalized eta squared can be used as an alternative for η² but not as an alternative for η²_w_.

In case of a design with a variable that is within participants and within stimuli, the correction to use is (observation – mean_participant_) + (observation – mean_stimulus_).
